# HIF-1/2α-Activated RNF146 Enhances the Proliferation and Glycolysis of Hepatocellular Carcinoma Cells via the PTEN/AKT/mTOR Pathway

**DOI:** 10.3389/fcell.2022.893888

**Published:** 2022-05-27

**Authors:** Guoliang Shen, Hao Wang, Ning Zhu, Qiliang Lu, Junwei Liu, Qiuran Xu, Dongsheng Huang

**Affiliations:** ^1^ Department of Clinical Medicine, Medical College of Soochow University, Suzhou, China; ^2^ Department of General Surgery, Zhejiang Provincial People’s Hospital, Affiliated People’s Hospital, Hangzhou Medical College, Hangzhou, China; ^3^ Department of Hepatobiliary Surgery, The First Affiliated Hospital of Xi’an Jiaotong University, Xi’an, China; ^4^ The Key Laboratory of Tumor Molecular Diagnosis and Individualized Medicine of Zhejiang Province, Zhejiang Provincial People’s Hospital, Affiliated People’s Hospital, Hangzhou Medical College, Hangzhou, China

**Keywords:** hepatocellular carcinoma, hypoxia microenvironment, RNF146, pten, akt/mtor pathway

## Abstract

Hypoxia microenvironment, a critical feature of hepatocellular carcinoma, contributes to hepatocarcinogenesis, tumor progression and therapeutic resistance. Hypoxia-inducible factors (HIFs)-activated target genes are the main effectors in hypoxia-induced HCC progression. In this study, we identified ubiquitin E3 ligase ring finger protein 146 (RNF146) as a novel HIFs target gene. Either HIF-1α or HIF-2α knockdown significantly repressed hypoxia-induced RNF146 upregulation in Hep3B and Huh7 cells. TCGA data and our immunohistochemistry analysis consistently revealed the overexpression of RNF146 in HCC tissues. The upregulated expression of RNF146 was also detected in HCC cell lines. The high RNF146 level was correlated with poor clinical features and predicted a shorter overall survival of patients with HCC. RNF146 knockdown suppressed the proliferation, colony formation and glycolysis of HCC cells, but suppressed but RNF146 overexpression promoted these malignant behaviors. Moreover, RNF146 silencing weakened HCC growth in mice. RNF146 inversely regulated phosphatase and tensin homolog (PTEN) protein level, thereby activating the AKT/mechanistic target of rapamycin kinase (mTOR) pathway in HCC cells. MG132 reversed RNF146 overexpression-induced PTEN reduction. RNF146 knockdown decreased the ubiquitination and degradation of PTEN in HCC cells. Therefore, we clarified that PTEN knockdown notably abolished the effects of RNF146 silencing on the AKT/mTOR pathway and Hep3B cells’ proliferation, colony formation and glycolysis. To conclude, our data confirmed that RNF146 was transcriptionally regulated by HIF-1/2α and activated the AKT/mTOR pathway by promoting the ubiquitin proteolysis of PTEN, thereby contributing to HCC progression. RNF146 may be a potential new drug target for anti-HCC.

## 1 Introduction

Hepatocellular carcinoma (HCC) accounts for 75–85% of all primary liver cancer cases and is the third leading cause of cancer-related death worldwide (Sung et al., 2021). In 2020, there are 910,000 new cases of liver cancer worldwide, of which 45% are from China (Sung et al., 2021). Surgical resection is still the best choice for HCC treatment, but most patients are diagnosed with advanced stage and lose the opportunity for surgery (Llovet et al., 2021a). The application of targeted therapy and immunotherapy has brought light to some patients with advanced HCC, but the overall prognosis has not improved significantly (Llovet et al., 2021a). Therefore, it is of great significance to further reveal the precise molecular mechanism of the occurrence and development of HCC and carry out new target discovery and intervention research of anti-cancer drugs to improve the curative effect and prognosis of patients.

Extracellular matrix deposition results in decreased blood flow to the liver, and rapid tumor growth, intense metabolic activity, and poor angiogenesis lead to the formation of a hypoxic microenvironment in HCC (Gilkes et al., 2014; Singleton et al., 2021). Multiple target genes transcriptionally regulated by hypoxia-inducible factors (HIFs) promote HSCs activation as well as the growth, metabolic reprogramming, stem cell-like properties, angiogenesis, invasion and metastasis of HCC (Liu et al., 2019; Cramer and Vaupel 2022). Hypoxia-induced ubiquitin-specific peptidase 13 (USP13) deubiquitinated and stabilized toll-like receptor 4 (TLR4) to activate the myeloid differentiation primary response gene 88/nuclear factor-κB (MyD88/NF-κB) pathway, thereby contributing to HCC progression (Gao et al., 2020). Peptidylarginine deiminase 4 (PADI4) is identified as a novel HIFs target gene and promotes the transcription activity of HIFs by inducing histone citrullination to enhance the growth and angiogenesis of HCC *in vivo* (Wang Y. et al., 2021). In addition to protein-coding genes, hypoxia affects microRNAs (miRNAs) and long non-coding RNAs (lncRNAs) to facilitate HCC progression. Hypoxia-induced miR-671–5p downregulation increases tuftelin1 (TUFT1) expression, promoting HCC growth and metastasis by activating the Ca^2+^/PI3K/AKT pathway (Dou et al., 2019). TM4SF1-AS1 is transcriptionally activated by HIF-1α and increases the migration, invasion and proliferation of HCC cells by upregulating the TM4SF1 level (Zeng et al., 2021). Therefore, further exploration of new hypoxia target genes is beneficial for revealing the new mechanism of hypoxia-induced HCC progression.

Ring finger protein 146 (RNF146), ubiquitin E3 ligase, belongs to the RING-type E3 ubiquitin ligase family (Darosa et al., 2015). RNF146 positively regulated the Wnt signaling pathway by affecting the protein degradation of AXIN1 (Zhang et al., 2011; Darosa et al., 2015; Matsumoto et al., 2017). RNF146 regulates the liver kinase B1 (LKB1)-AMP-activated protein kinase (AMPK) pathway by mediating LKB1 ubiquitination (Li et al., 2019). Phosphatase and tensin homolog (PTEN) is recognized as a protein substrate of RNF146. RNF146 results in the ubiquitination and degradation of PTEN to activate the AKT pathway and promotes tumor cell proliferation and glycolysis (Li et al., 2015). In HCC, RNF146 is the regulator of poly (ADP-ribose) polymerase 1 (PARP1) ubiquitination and protein degradation (Zhou et al., 2020). However, the link between hypoxia and RNF146 in HCC remains unclear yet.

In this study, we explore candidate HIFs target genes by analyzing microarray data. The regulatory effects of HIF-1/2α on RNF146 expression were confirmed. The role of RNF146 in HCC cell proliferation and glycolysis, as well as underlying mechanisms, were investigated. We found that hypoxia enhanced RNF146 expression via HIF-1/2α. RNF146 contributed to the proliferation, colony formation and glycolysis of HCC cells by regulating the PTEN/AKT/mechanistic target of rapamycin kinase (mTOR) pathway.

## 2 Materials and Methods

### 2.1 Tissue Samples

This study collected human HCC tissue samples and paired adjacent tissue samples from 80 patients with HCC in the First Affiliated Hospital of Xi’an Jiaotong University. The included patients had precise pathological diagnoses and had not been treated before surgery. All the collected tissue samples were well were formalin-fixed and paraffin-embedded. Written informed consent was obtained from all patients, and the Ethics Committee of the 1st Affiliated Hospital of Xi’an Jiaotong University approved this study. During 3 years of follow-up, the time between primary surgery and death was the overall survival time.

### 2.2 Cell Culture

Hep3B, HepG2, HCCLM3 and Huh7 cells were provided by Stem Cell Bank, Chinese Academy of Sciences (Shanghai, China). Human liver cell line MIHA was purchased from bnbio (Beijing, China). Cell lines were cultured in DMEM (Gibco, Thermo Fisher Scientific, Waltham, MA, United States) added with 10% fetal bovine serum (Gibco), penicillin (100 U/ml, Gibco) and streptomycin (100 mg/ml, Gibco). The culture condition in the incubator was a moist environment with 5% CO_2_ at 37°C.

### 2.3 Lentivirus and Plasmid Construction

LKO.1-puro lentiviral vectors encoding small hairpin RNA (shRNA) against HIF-1α (sh1α), HIF-2α (sh2α), RNF146 (shRNF#1 and shRNF#2), PTEN (shPTEN) and non-targeting shRNA (NTC) were provided by GeneChem (Shanghai, China). All lentiviral shuttle vectors were transfected into HEK293T cells for packaging as previously described (Zeng et al., 2021). RNF146 cDNA was sub-cloned into pcDNA3.1 (Invitrogen, Carlsbad, CA, United States) to produce pcDNA3.1/RNF146 (RNF-OE). Lipofectamine 3000 (Thermo Fisher Scientific) was applied for plasmid transfection in HCC cells.

### 2.4 Real-Time Quantitative PCR

HCC cells were subjected to RNA isolation following the instruction of the Trizol reagent (Invitrogen) and total RNA was reversely transcribed into cDNA using PrimeScript™ RT Master Mix (Takara, Shiga, Japan). The PCR amplification was performed on ABI HT9600 (Applied Biosystems, Foster City, CA, United States) in accordance with the instruction of SYBR Green PCR Master Mix (Takara). The RNF146 mRNA level was normalized to GAPDH using the 2^−ΔΔCT^ method. RNF146 forward primer: 5′-TGT AAG CAC GTT TTC TGC TAT CT-3′; reverse: 5′-AAT CCT CGG GAA TTT CTT GTC G-3′. GAPDH forward primer: 5′-CTG GGC TAC ACT GAG CAC C-3′; reverse: 5′-AAG TGG TCG TTG AGG GCA ATG-3′.

### 2.5 Western Blotting

Total proteins were obtained from HCC cells using RIPA buffer (Beyotime, Shanghai, China) and quantified by Enhanced BCA Protein Assay Kit (Beyotime). Then, 20 μg proteins were undergoing SDS-PAGE and transferred to the PVDF membrane (Millipore, Billerica, MA, United States). After blocking, the membrane was incubated overnight with primary antibodies at 4°C. The next day, secondary antibody incubation was performed at room temperature for 1–2 h. Enhanced chemiluminescence (ECL) reagent (Millipore) was used for luminous reaction and western blot was photographed by Amersham Imager 680 (GE Healthcare Life Sciences, Pittsburgh, PA, United States). The used primary antibodies: HIF-1α (ab1, Abcam, Cambridge, MA, United States), HIF-2α (ab199, Abcam), RNF146 (ab201212, Abcam), *β*-tubulin (10094-1-AP, Proteintech, Wuhan, China), PTEN (ab267787, Abcam). p-AKT (Ser473, 66444-1-Ig, Proteintech), AKT (60203-2-Ig, Proteintech), p-mTOR (Ser2448, 67778-1-Ig, Proteintech), mTOR (66888-1-Ig, Proteintech), Ubiquitin (ab7254, Abcam).

### 2.6 Immunohistochemistry

The embedded-paraffin tissues were sectioned with a thickness of approximately 4–5 μm. Next, paraffin sections were deparaffinized in xylene and rehydrated with ethanol, and used 0.3% hydrogen peroxide to block endogenous peroxidase activity. Finally, the sections were incubated with RNF146 antibody (ab201212, Abcam) and a specific secondary antibody. The intensity score and positive rate score were previously described (Shen et al., 2018). The IHC score (intensity score × positive rate score) ≥4 was defined as high expression.

### 2.7 Immunoprecipitation and Ubiquitination Assays

The immunoprecipitation and ubiquitination assays were performed as previously described (Shi et al., 2021). In brief, the cells were incubated with 10 μM MG132 (HY-13259, MedChemExpress, Shanghai, China) for 8 h. HCC cells were treated with IP lysis buffer. The total cell lysates were collected and subjected to immunoprecipitation with 2 µg PTEN (ab267787, Abcam) or IgG antibody and 30 µL slurry of Protein G Sepharose (GE Healthcare Life Sciences, Pittsburgh, PA, United States) at 4°C overnight. Western blot analysis was performed with an Anti-Ubiquitin antibody (ab7254, Abcam) to detect the corresponding protein ubiquitination.

### 2.8 Cell Proliferation and Colony Formation Assays

The cell counting kit-8 (CCK-8) kit (Beyotime) and Cell-Light™ EdU Apollo^®^488 *In Vitro* Imaging Kit (RIBOBIO, Guangzhou, China) were used for HCC cell proliferation determination as previously described (Shi et al., 2021). For colony formation assay, the logarithmic growth cells (1 × 10^3^) were seeded into 6-well plates and were grown for a period of 2 weeks. Cell colonies were stabilized with 4% paraformaldehyde, stained with 0.1% crystal violet solution and manually counted.

### 2.9 Glucose Consumption and Lactate Production Assays

Glucose consumption and lactate production of HCC cells were detected using the lactate assay kit (ab65331, Abcam) and glucose assay kit (ab136955, Abcam), respectively, according to the manufacturer’s protocols.

### 2.10 *In vivo* Studies

The animal studies were approved by the Institutional Animal Care and Use Committee of Xi’an Jiaotong University. Male BALB/c nude mice (4 weeks old) were obtained from the Vital River Laboratory Animal Technology Co. (Beijing, China). 5 × 10^6^ HCCLM3 cells stably transfected with RNF146 shRNA or the negative control were respectively injected into the right armpits of five mice, and subcutaneous tumor size was assessed every week. The mice were sacrificed 4 weeks after injection and the xenograft tumors were removed for IHC with Ki-67 antibody (27309-1-AP, Proteintech) or WB with RNF146 antibody (ab201212, Abcam).

### 2.11 Statistical Analysis

All data in this study were processed by GraphPad Prism 8 (San Diego, CA, United States). Measurement data were shown as mean ± S.D. The Mann–Whitney test and analysis of variance (ANOVA) were applied for the significant test. Survival data were analyzed through the Kaplan-Meier method and log-rank test. *p*-value less than 0.05 denoted statistically significant.

## 3 Results

### 3.1 Hypoxia-Inducible Factor-1/2α Regulates RNF146 Expression in Hepatocellular Carcinoma Cells Under Hypoxic Conditions

**FIGURE 1:**
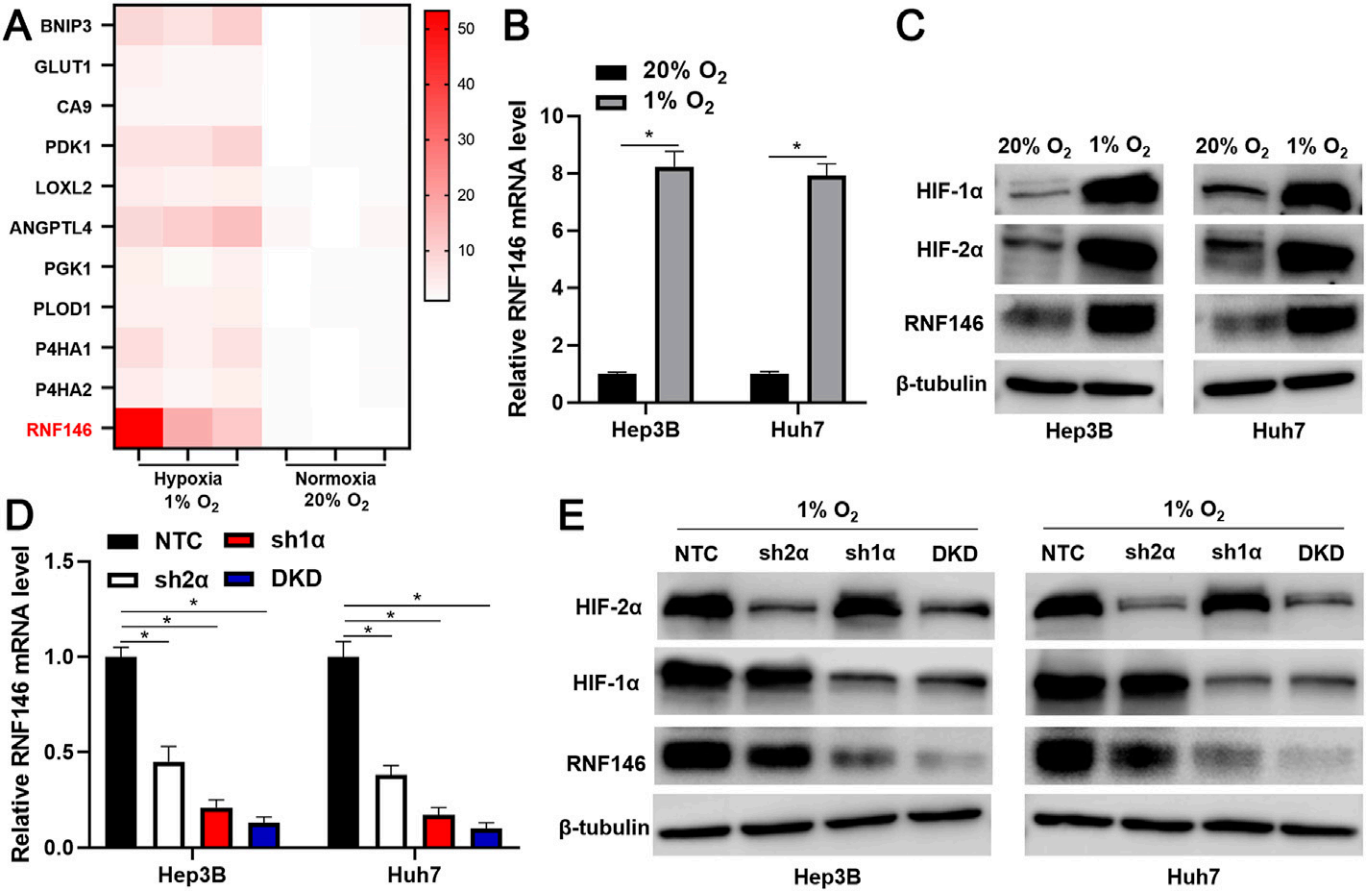
RNF146 is a hypoxia-responsive gene. **(A)** Heatmap of ten known hypoxia target genes and RNF146 expression in Hep3B cells under normoxia (20% O_2_) and hypoxia (1% O_2_) conditions. **(B)** The expression of RNF146 mRNA in HCC cells under normoxia and hypoxia conditions was measured by RT-qPCR. **(C)** HIF-1α, HIF-2α and RNF146 protein levels were determined by WB in HCC cells under normoxia and hypoxia conditions. **(D)** The lentivirus-mediated shRNAs against HIF-2α (sh2α), HIF-1α (sh1α), HIF-1α/2α (DKD) and non-targeting shRNA (NTC) were transduced into HCC cells. After incubation under hypoxia conditions, RNF146 mRNA level in HCC cells was measured by RT-qPCR. **(E)** HIF-1α, HIF-2α and RNF146 protein levels were determined by WB in transfected HCC cells under hypoxia conditions. *p < 0.05.

We analyzed our previous microarray data (GSE155505) of hypoxia-related mRNAs in Hep3B cells. A significant upregulation of RNF146 in Hep3B cells under hypoxic conditions caught our attention (Figure 1A). RT-qPCR and WB analyses consistently confirmed that hypoxia upregulated RNF146 expression in Huh7 and Hep3B cells (*p* < 0.05, Figures 1B,C). Then, shRNAs were used to downregulate HIF-1α or HIF-2α or HIF1/2α in HCC cells (Figure 1E). The silencing of HIF-1α or HIF-2α or both [double knockdown (DKD)] prominently reduced hypoxia-induced RNF146 expression in HCC cells (*p* < 0.05, Figures 1D,E). Furthermore, TCGA data analysis using the GEPIA website (http://gepia.cancer-pku.cn/) (Tang et al., 2019) found positive correlations between RNF146 and HIF1/2α in HCC tissues (*p* < 0.05, Supplementary Figure S1). Our data suggest that hypoxia induces RNF146 expression via HIF-1/2α in HCC cells.

### 3.2 RNF146 is Highly Expressed in Hepatocellular Carcinoma

**FIGURE 2:**
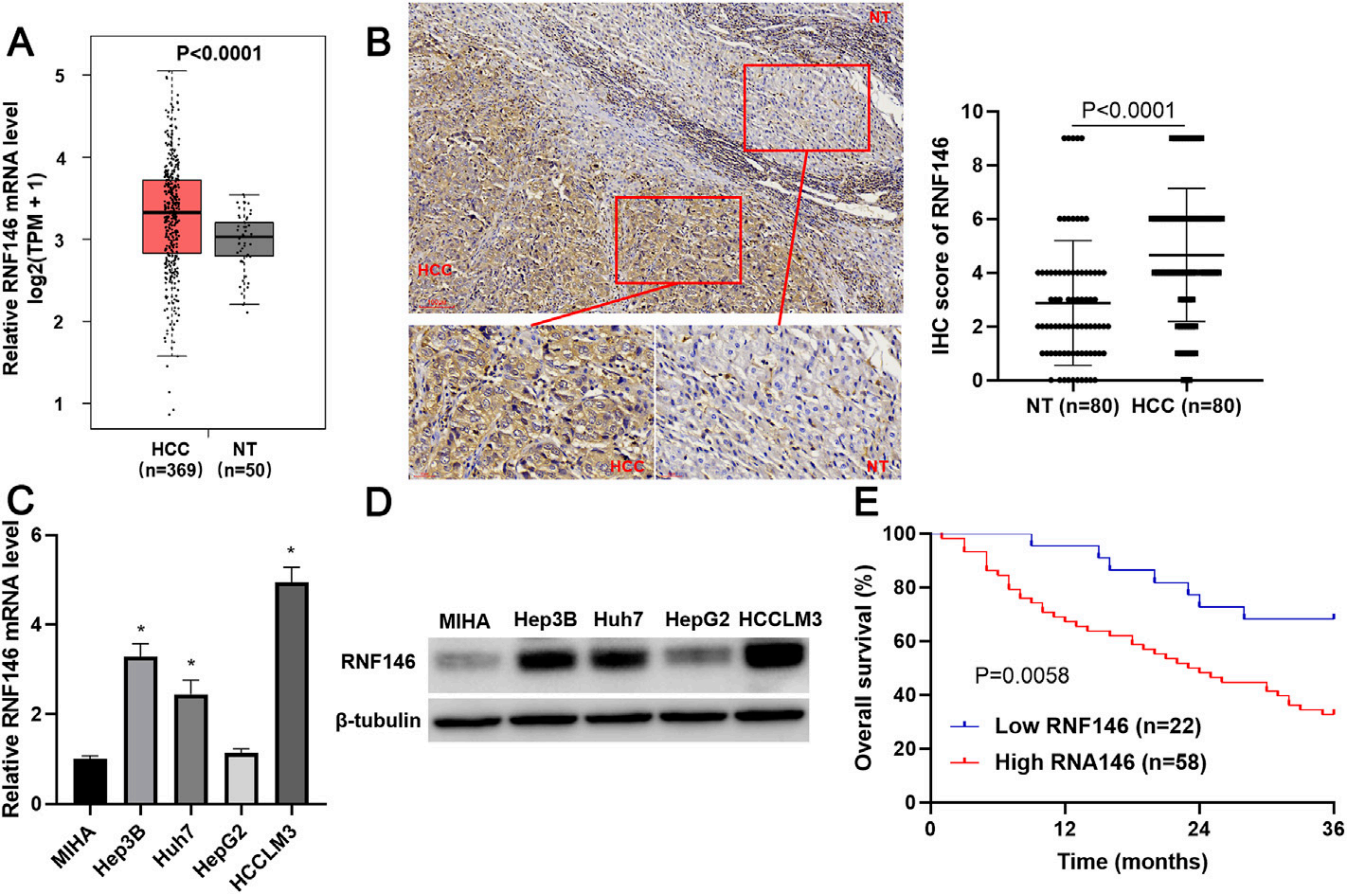
RNF146 expression is increased in HCC. **(A)** TCGA data analysis using the GEPIA platform showed the expression difference of RNF146 mRNA between HCC and normal liver tissues. **(B)** IHC analysis indicated that RNF146 was highly expressed in HCC compared to adjacent nontumor tissues. **(C)** The RNF146 mRNA levels were detected in MIHA, Hep3B, Huh7, HepG2 and HCCLM3 cells by RT-qPCR. **(D)** The RNF146 protein levels were detected in MIHA, Hep3B, Huh7, HepG2 and HCCLM3 cells by WB. **(E)** The comparison of 3-years overall survival of patients with high or low RNF146 expression.

TCGA data analysis using the GEPIA website (http://gepia.cancer-pku.cn/) (Tang et al., 2019) found that RNF146 mRNA expression in HCC was prominently higher than in normal liver tissues (*p* < 0.0001, Figure 2A). IHC data confirmed the upregulated level of RNF146 protein in HCC tissues compared to adjacent nontumor tissues (*p* < 0.0001, Figures 2B,C). Moreover, the upregulated levels of RNF146 were also detected in Hep3p, Huh7 and HCCLM3 cells (*p* < 0.05, Figure 2D). Table 1 showed that HCC patients with large tumors and venous infiltration expressed higher levels of RNF146 (*p* < 0.05). Survival analysis indicated a close correlation between high RNF146 expression and poor 3-years overall survival of patients with HCC (*p* = 0.0058, Figure 2E). Thus, these results indicate that RNF146 may be a promising prognostic biomarker for HCC.

**TABLE 1:** Relationship between RNF146 expression and clinicopathologic parameters of patients with hepatocellular carcinoma.

Clinicopathologic Parameters		*n* = 80	RNF146		*p*
			Low Expression (n = 22)	High Expression (*n* = 58)	
Age (years)	<50	33	10	23	0.638
	≥50	47	12	35	
Sex	Male	67	18	49	0.773
	Female	13	4	9	
HBV infection	No	13	5	8	0.333
	Yes	67	17	50	
Serum AFP level (ng/ml)	<20	14	6	8	0.157
	≥20	66	16	50	
Tumor size (cm)	<5	29	12	17	0.036^a^
	≥5	51	10	41	
No. of tumor nodules	1	65	20	45	0.173
	≥2	15	2	13	
Cirrhosis	No	12	5	7	0.233
	Yes	68	17	51	
Venous infiltration	No	44	17	27	0.014^a^
	Yes	36	5	31	
Tumor differentiation	I + II	53	16	37	0.451
	III + IV	27	6	21	
TNM stage	I	32	12	20	0.102
	II + III	48	10	38	
BCLC stage	0 + A	59	19	40	0.114
	B + C	21	3	18	

HBV, hepatitis B virus; AFP, alpha-fetoprotein; TNM, tumor-node-metastasis; BCLC, Barcelona Clinic Liver Cancer.

a Statistically significant.

### 3.3 RNF146 Promotes Hepatocellular Carcinoma Cell Proliferation and Glycolysis

**FIGURE 3:**
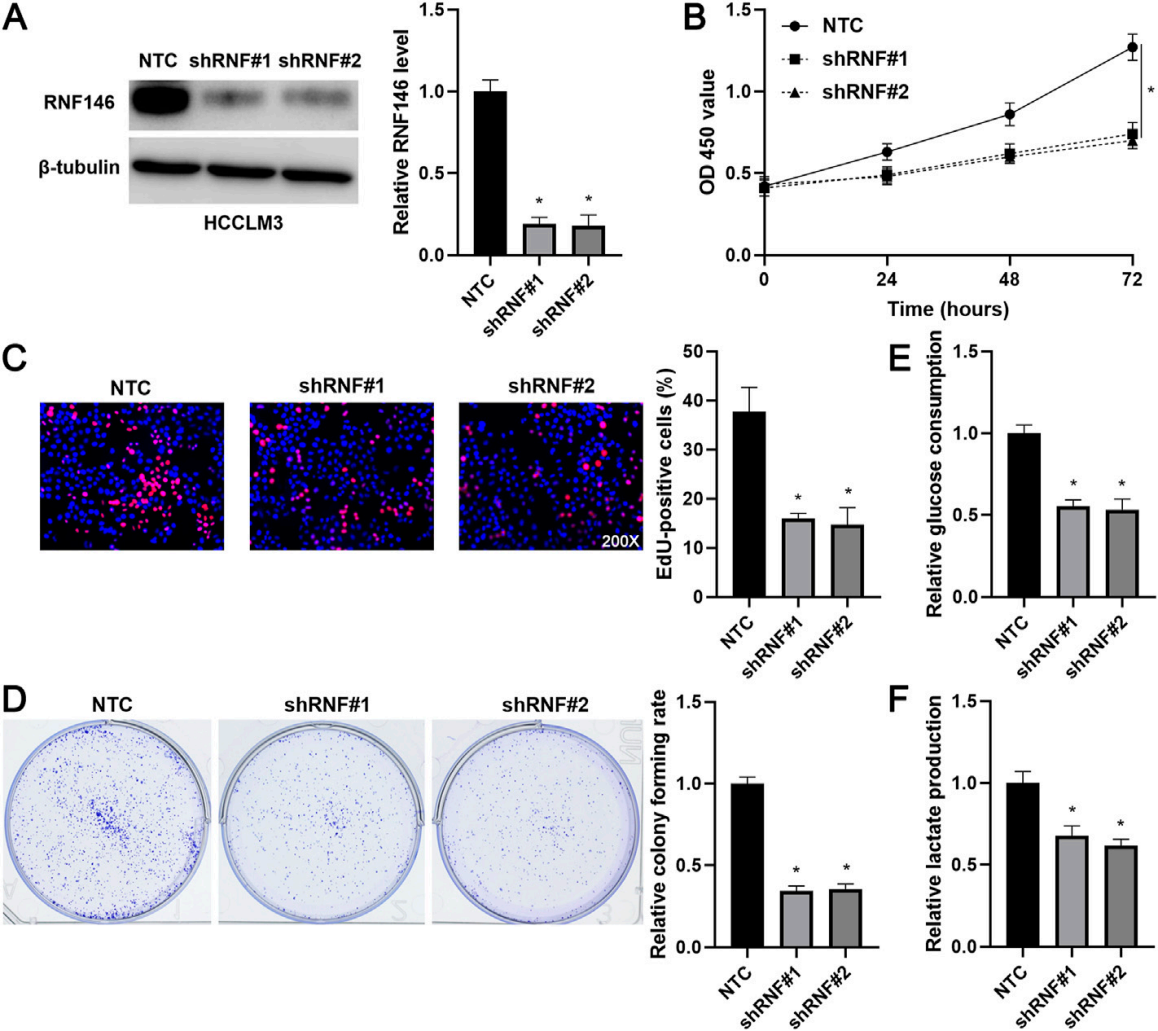
RNF146 knockdown suppresses the proliferation and glycolysis of HCCLM3 cells. **(A)** The lentivirus-mediated shRNAs targeting RNF146 (shRNF#1 and shRNF#2) and non-targeting shRNA (NTC) were transduced into HCCLM3 cells. WB was performed to determine the RNF146 protein level. **(B)** RNF146 knockdown reduced the viability of HCCLM3 cells. **(C)** RNF146 silencing inhibited the proliferation of HCCLM3 cells. **(D)** The inhibitory effects of RNF146 knockdown on HCCLM3 cell colony formation were confirmed. **(E)** RNF146 silencing reduced the glucose consumption of HCCLM3 cells. **(F)** HCCLM3 cells’ lactate production was decreased by RNF146 knockdown. *p < 0.05.

**FIGURE 4:**
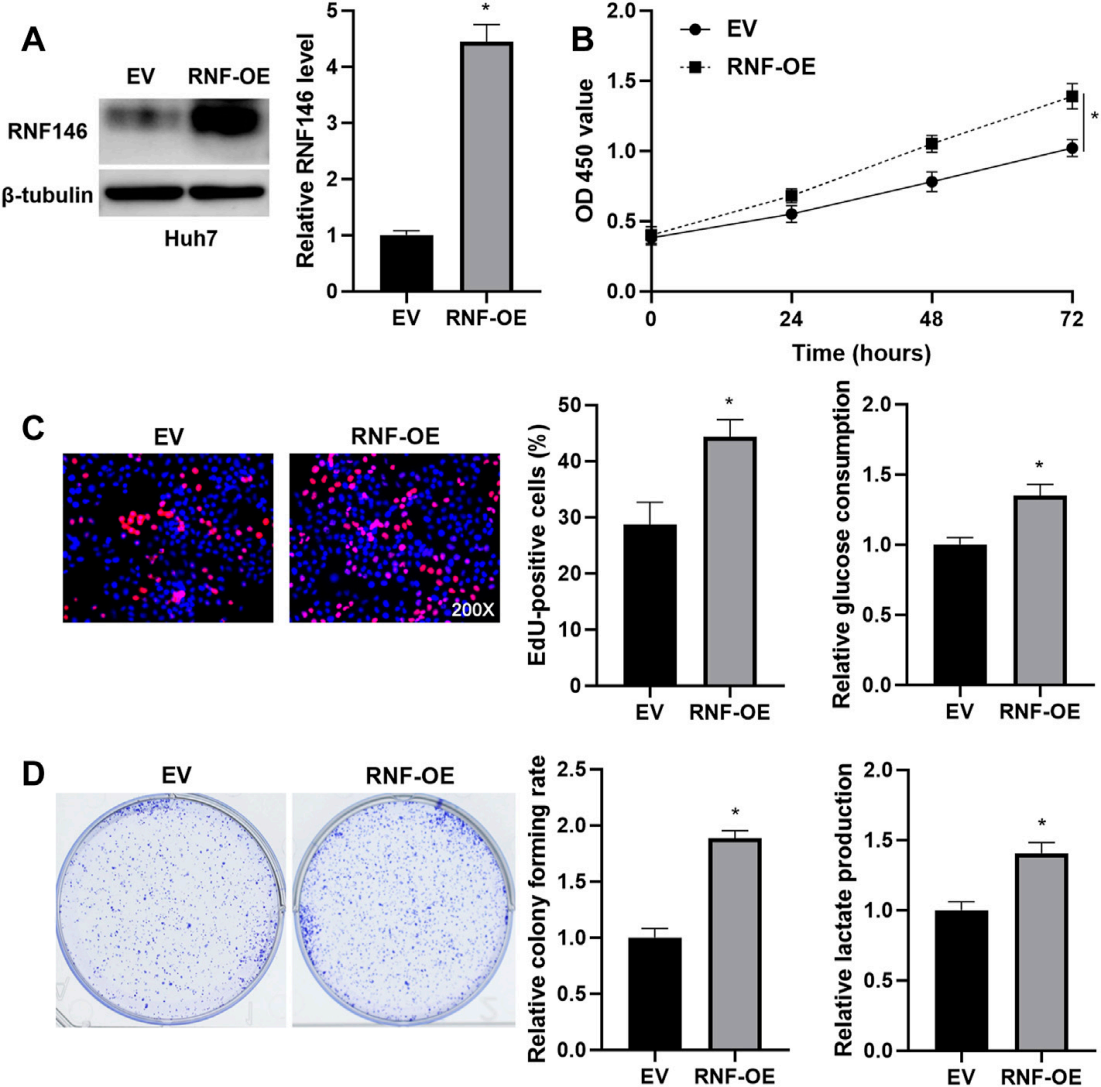
RNF146 overexpression promotes the proliferation and glycolysis of Huh7 cells. **(A)** The pcDNA3.1/RNF146 (RNF-OE) and empty vector (EV) were transduced into Huh7 cells. WB was performed to determine the RNF146 protein level. **(B)** CCK-8, **(C)** EdU, **(D)** colony formation, E glucose consumption and F lactate production assays were performed to detect the proliferation, colony formation and glycolysis of Huh7 cells with or without RNF146 overexpression. *p < 0.05.

**FIGURE 5:**
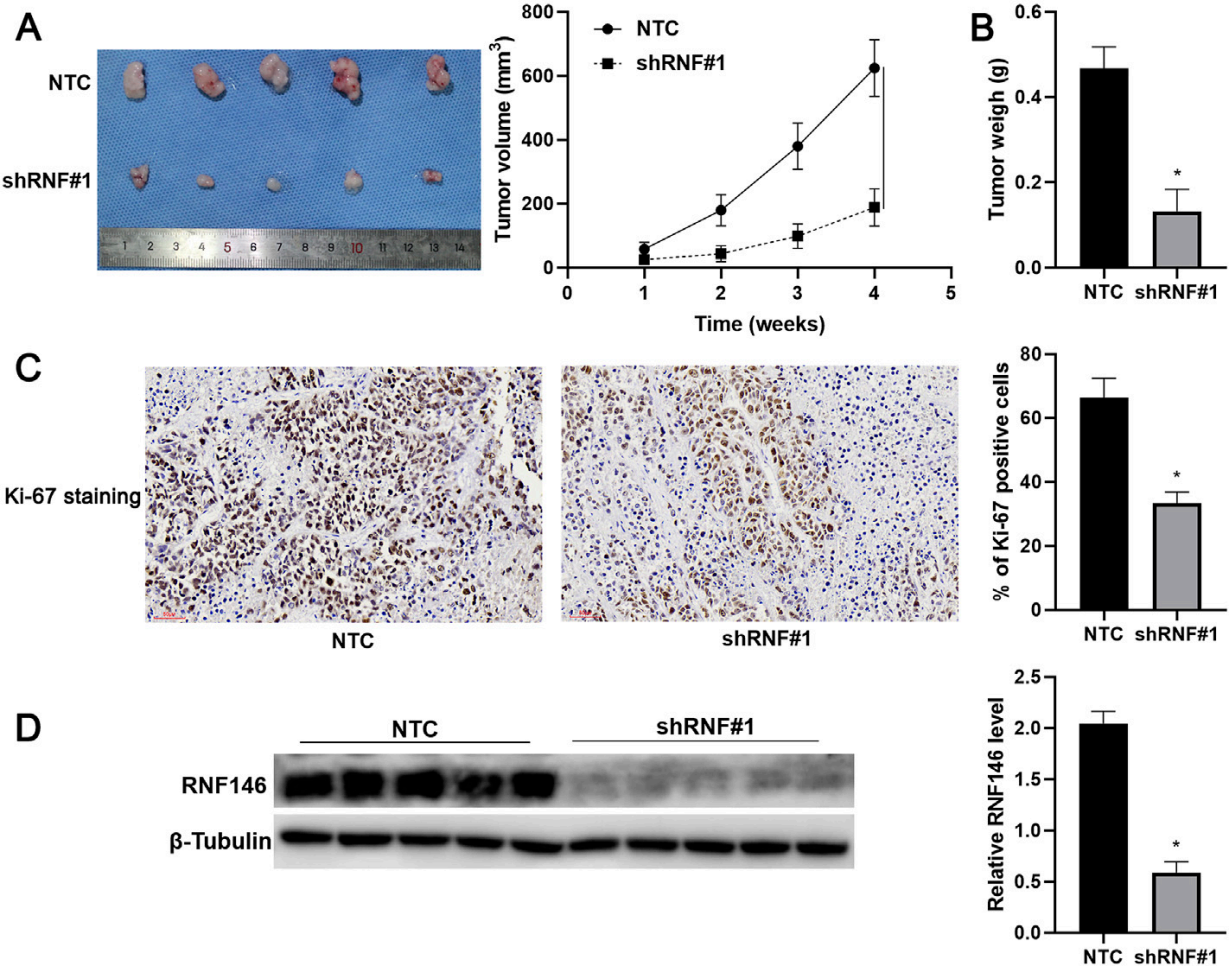
RNF146 knockdown reduces HCC growth *in vivo*. HCCLM3 cells with or without RNF146 knockdown were subcutaneously injected into nude mice. **(A)** Tumor volume and **(B)** weight were consistently reduced by RNF146 knockdown. **(C)** Tumor tissues from the RNF146 knockdown group showed fewer Ki-67 staining cells compared to the control group. **(D)** Tumor tissues from the RNF146 knockdown group showed lower RNF146 protein levels than the control group. *p < 0.05.

Lentivirus-mediated shRNAs markedly downregulated RNF146 in HCCLM3 cells (*p* < 0.05, Figure 3A). RNF146 knockdown remarkably reduced the viability of HCCLM3 cells (*p* < 0.05, Figure 3B). The silencing of RNF146 significantly weakened the proliferation of HCCLM3 cells (*p* < 0.05, Figure 3C). The HCCLM3 cell colonies were obviously decreased by RNF146 silencing (*p* < 0.05, Figure 3D). Otherwise, RNF146 depletion remarkably decreased the glucose consumption and lactate production of HCCLM3 cells (*p* < 0.05, Figures 3E,F). RNF146 overexpression prominently enhanced the proliferation and glycolysis of Huh7 cells (*p* < 0.05, Figure 4). Next, *in vivo* experiments confirmed that RNF146 knockdown reduced the volume and weight of subcutaneous tumors formed by HCCLM3 cells (*p* < 0.05, Figures 5A,B). IHC staining of Ki-67 in tumor tissues indicated that the percentage of positive cells in the RNF146 knockdown group was obviously lower than in the control group (*p* < 0.05, Figure 5C). WB analysis confirmed the downregulated expression of RNF146 in the RNF146 knockdown group compared to the control group (*p* < 0.05, Figure 5D). Collectively, we consider RNF146 as an oncogene in HCC.

### 3.4 RNF146 Regulates the AKT/mTOR Pathway by Reducing Phosphatase and Tensin Homolog

**FIGURE 6:**
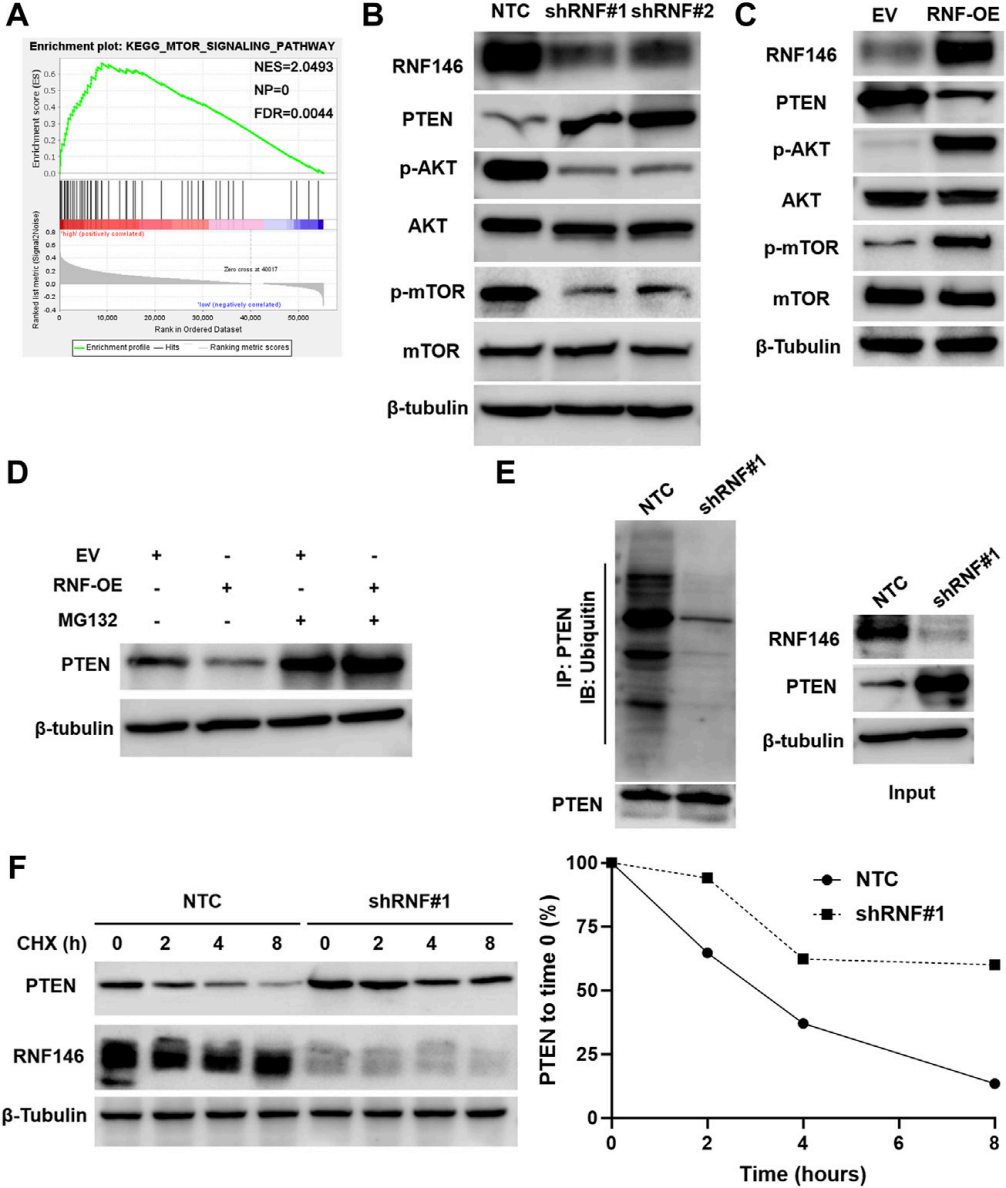
RNF146 regulates the PTEN/AKT/mTOR pathway in HCC cells. **(A)** KEGG pathway enrichment analysis indicated a close correlation between RNF146 and the mTOR signaling pathway in HCC. **(B)** RNF146 knockdown increased PTEN expression but reduced p-AKT (Ser473) and p-mTOR (Ser2448) levels in HCCLM3 cells. **(C)** RNF146 overexpression decreased PTEN expression while increased p-AKT (Ser473) and p-mTOR (Ser2448) levels in Huh7 cells. **(D)** RNF146 overexpression-induced PTEN downregulation was blocked by a proteasome inhibitor MG132 in Huh7 cells. **(E)** The ubiquitination of PTEN was reduced by RNF146 silencing in HCCLM3 cells. **(F)** The degradation of PTEN was reduced by RNF146 silencing in HCCLM3 cells.

KEGG enrichment pathways analysis suggested that RNF146 might regulate the mTOR signaling pathway in HCC based on TCGA data (Figure 6A and Supplementary Table S1). RNF146 knockdown decreased while RNF146 overexpression increased p-AKT and p-mTOR levels in HCC cells (Figures 6B,C). A previous study has identified RNF146 as a negative regulator of PTEN and activates the PI3K/AKT/mTOR pathway (Li et al., 2015). Interestingly, we found that RNF146 inversely regulated PTEN protein levels in HCC cells (Figures 6B,C). A proteasome inhibitor, MG132, blocked RNF146-induced PTEN downregulation in Huh7 cells (Figure 6D). Moreover, RNF146 silencing obviously reduced the ubiquitination and degradation of PTEN in HCC cells (Figures 6E,F). Therefore, RNF146 promotes PTEN ubiquitination and degradation and activates the AKT/mTOR pathway in HCC.

### 3.5 The Role of RNF146 Silencing is Reversed by Phosphatase and Tensin Homolog Knockdown in Hepatocellular Carcinoma Cells

**FIGURE 7:**
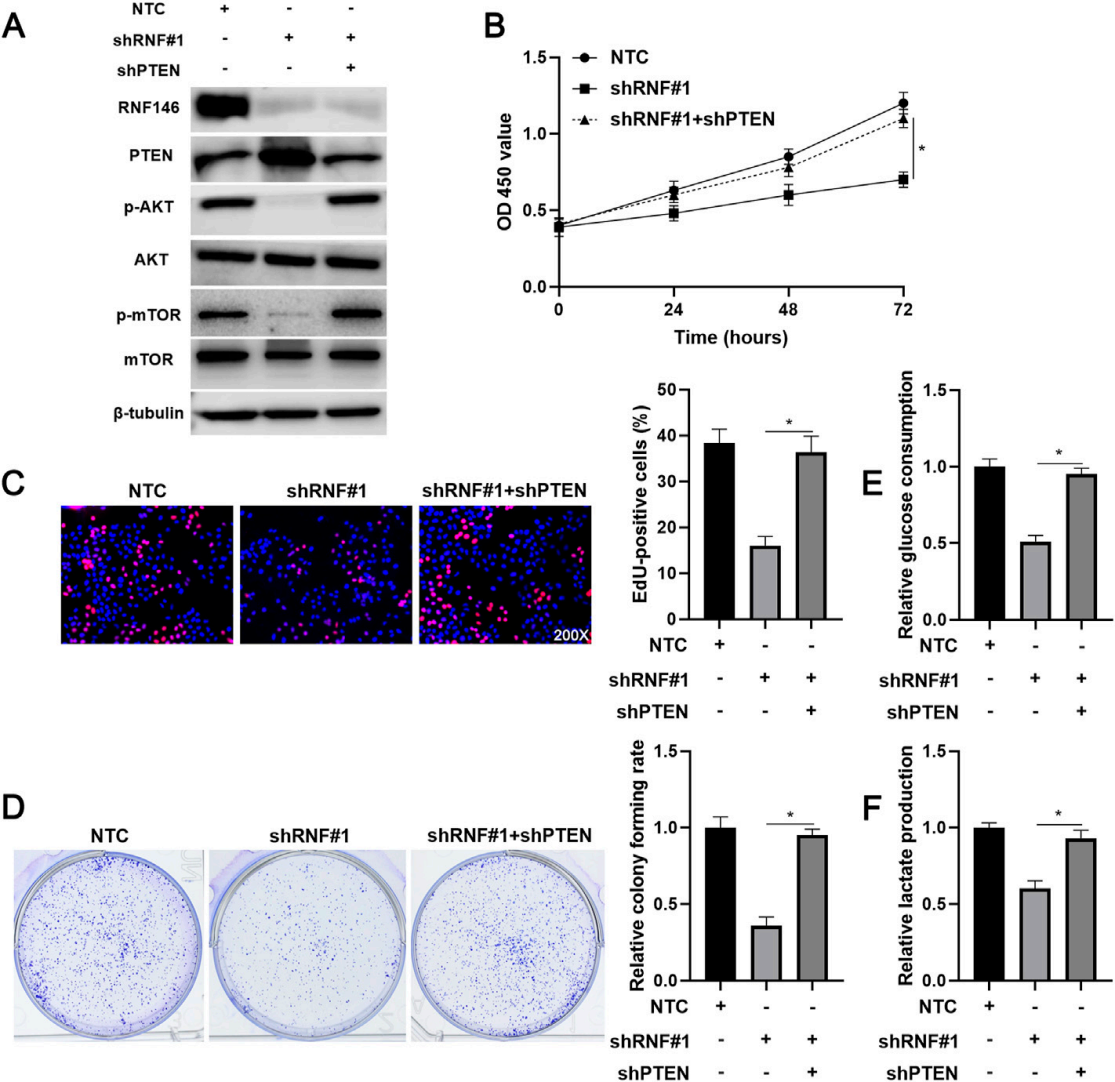
Reducing PTEN expression abolishes the effects of RNF146 knockdown in HCCLM3 cells. **(A)** HCCLM3 cells that were transfected with NTC, shRNF#1 or shRNF#1 + shPTEN were detected by WB for the RNF146, PTEN, p-AKT (Ser473), AKT, p-mTOR (Ser2448) and mTOR levels. **(B)** CCK-8, **(C)** EdU, **(D)** colony formation, **(E)** glucose consumption and **(F)** lactate production assays were performed to detect the proliferation, colony formation and glycolysis of HCCLM3 cells with or without RNF146 overexpression. *p < 0.05.

We intended to verify the involvement of PTEN in RNF146-induced HCC progression. A specific shRNA downregulated PTEN expression in HCCLM3 cells with RNF146 knockdown (Figure 7A). PTEN knockdown recovered RNF146 silencing-induced p-AKT and p-mTOR downregulation in HCCLM3 cells (Figure 7A). We confirmed that PTEN silencing significantly abolished RNF146 knockdown-induced proliferation inhibition of HCCLM3 cells (*p* < 0.05, Figures 7B,C). Moreover, the effects of RNF146 silencing on HCCLM3 cell colony formation, glucose consumption and lactate production were markedly reversed by PTEN knockdown (*p* < 0.05, Figures 7D–F). Therefore, PTEN mediates the pro-HCC function of RNF146.

## 4 Discussion

Hypoxia is one of the core features of the HCC microenvironment and a key factor leading to tumor malignant progression and treatment resistance (Jing et al., 2019). Monoclonal antibodies (bevacizumab and ramucirumab) and small-molecule inhibitors (sorafenib and lenvatinib) targeting the hypoxia-responsive gene VEGF and its receptors have become first-line treatments for advanced HCC (Llovet et al., 2021b). In this study, we aimed to investigate novel hypoxia-responsive genes in HCC. According to microarray data analysis, we selected a hypoxia-upregulated gene, RNF146, for further study. The expression of RNF146 was prominently upregulated in HCC cells under hypoxia conditions. The knockdown of HIF-1α or HIF-2α or DKD significantly blocked hypoxia-induced RNF146 upregulation in HCC cells. These results suggest that RNF146 is a new HIF-1/2α target gene.

RNF146 overexpression is frequently detected in colorectal cancer (CRC) and is an independent biomarker for predicting the poor prognosis (Shen et al., 2018). RNF146 expression is upregulated in non-small cell lung cancer (NSCLC) and is associated with malignant clinical features and poor clinical outcomes (Gao et al., 2014). Here, we also verified that RNF146 was highly expressed in HCC based on TCGA data and our IHC data. The upregulated levels of RNF146 were consistently determined in HCC cell lines. Moreover, we found that high RNF146 expression was correlated with large tumors and venous infiltration and predicted a shorter 3-years overall survival of patients with HCC. Our data verify the potential clinical value of RNF146 in the judgment of poor prognosis in HCC.

RNF146 knockdown repressed the proliferation, colony formation and glycolysis of HCC cells, while RNF146 overexpression facilitated these malignant behaviors. RNF146 silencing reduced HCC growth in nude mice. These data recognize RNF146 as an oncogene in HCC. Therefore, we further explored the possible molecular mechanism of RNF146 promoting HCC. Previous studies have demonstrated that RNF146 activates the Wnt/β-catenin pathway by mediating ubiquitination and degradation of AXIN1 (Zhang et al., 2011; Gao et al., 2014; Shen et al., 2018). RNF146 modulates the LKB1-AMPK pathway by promoting LKB1 ubiquitination (Li et al., 2019). RNF146 mediates the ubiquitination and degradation of PTEN to activate the AKT pathway and promotes tumor cell proliferation and glycolysis (Li et al., 2015). RNF146 is a regulator of PARP1 ubiquitination and protein degradation in HCC (Zhou et al., 2020). Here, KEGG pathway analysis based on TCGA data showed that the mTOR pathway is one of the most relevant signaling pathways for RNF146. Next, we confirmed that RNF146 promoted the AKT/mTOR pathway in HCC cells. RNF146 overexpression-induced PTEN downregulation was reversed by MG132 treatment. RNF146 silencing increased the ubiquitination and degradation of PTEN in HCC cells. Thus, we consider that RNF146 regulates the AKT/mTOR pathway by ubiquitinating and degrading PTEN in HCC. The PTEN/AKT/mTOR pathway is one of the critical oncogenic pathways in HCC and participates in the regulation of cell proliferation, glycolysis, stemness and invasion (Liu et al., 2018; Dou et al., 2019; Dou et al., 2020; Wang L. et al., 2021). PTEN knockdown recovered RNF146 silencing-induced the AKT/mTOR pathway inactivation in HCCLM3 cells. Significantly, reducing PTEN expression prominently abolished the effects of RNF146 knockdown in HCC cells. Thus, RNF146 contributes to HCC progression by regulating the PTEN/AKT/mTOR pathway.

Our findings identified RNF146 as a novel HIF-1/2α target gene in HCC. HIF-1/2α-activated RNF146 facilitated HCC progression by enhancing PTEN ubiquitination and degradation and promoting the AKT/mTOR pathway activation. This study provides a new idea for developing new targets for anti-HCC drugs.

## Data Availability

The datasets presented in this study can be found in online repositories. The names of the repository/repositories and accession number(s) can be found below: https://www.ncbi.nlm.nih.gov/geo/, GSE155505;https://portal.gdc.cancer.gov/, TCGA-LIHC.

## Glossary

AMPK,: AMP-activated protein kinase
CRC,: colorectal cancer
HCC: hepatocellular carcinoma
HIFs,: hypoxia-inducible factors
LKB1,: liver kinase B1
lncRNAs,: long non-coding RNAs
miRNAs,: microRNAs
mTOR,: mechanistic target of rapamycin kinase
MyD88,: myeloid differentiation primary response gene 88
NF-κB,: nuclear factor-κB
NSCLC,: non-small cell lung cancer.
PADI4,: peptidylarginine deiminase 4
PARP1,: poly (ADP-ribose) polymerase 1
PTEN,: phosphatase and tensin homolog
RNF146,: ring finger protein 146
TLR4,: toll-like receptor 4
TUFT1,: tuftelin1
USP13,: ubiquitin specific peptidase 13

## References

[B1] Cramer T. Vaupel P. (2022). Severe Hypoxia Is a Typical Characteristic of Human Hepatocellular Carcinoma: Scientific Fact or Fallacy? J. Hepatol. 76 (4), 975–980. 10.1016/j.jhep.2021.12.028 34990751

[B2] Darosa P. A. Wang Z. Jiang X. Pruneda J. N. Cong F. Klevit R. E. (2015). Allosteric Activation of the RNF146 Ubiquitin Ligase by a poly(ADP-Ribosyl)ation Signal. Nature 517, 223–226. 10.1038/nature13826 25327252 PMC4289021

[B3] Dou C. Sun L. Wang L. Cheng J. Wu W. Zhang C. (2020). Bromodomain-containing Protein 9 Promotes the Growth and Metastasis of Human Hepatocellular Carcinoma by Activating the TUFT1/AKT Pathway. Cell Death Dis. 11, 730. 10.1038/s41419-020-02943-7 32908135 PMC7481201

[B4] Dou C. Zhou Z. Xu Q. Liu Z. Zeng Y. Wang Y. (2019). Hypoxia-induced TUFT1 Promotes the Growth and Metastasis of Hepatocellular Carcinoma by Activating the Ca2+/PI3K/AKT Pathway. Oncogene 38, 1239–1255. 10.1038/s41388-018-0505-8 30250300

[B5] Gao S. Chen T. Li L. Liu X. Liu Y. Zhao J. (2020). Hypoxia-Inducible Ubiquitin Specific Peptidase 13 Contributes to Tumor Growth and Metastasis via Enhancing the Toll-like Receptor 4/Myeloid Differentiation Primary Response Gene 88/Nuclear Factor-Κb Pathway in Hepatocellular Carcinoma. Front. Cell Dev. Biol. 8, 587389. 10.3389/fcell.2020.587389 33195243 PMC7604352

[B6] Gao Y. Song C. Hui L. Li C.-y. Wang J. Tian Y. (2014). Overexpression of RNF146 in Non-small Cell Lung Cancer Enhances Proliferation and Invasion of Tumors through the Wnt/β-Catenin Signaling Pathway. PLoS One 9, e85377. 10.1371/journal.pone.0085377 24454854 PMC3891871

[B7] Gilkes D. M. Semenza G. L. Wirtz D. (2014). Hypoxia and the Extracellular Matrix: Drivers of Tumour Metastasis. Nat. Rev. Cancer 14, 430–439. 10.1038/nrc3726 24827502 PMC4283800

[B8] Jing X. Yang F. Shao C. Wei K. Xie M. Shen H. (2019). Role of Hypoxia in Cancer Therapy by Regulating the Tumor Microenvironment. Mol. Cancer 18, 157. 10.1186/s12943-019-1089-9 31711497 PMC6844052

[B9] Li N. Wang Y. Neri S. Zhen Y. Fong L. W. R. Qiao Y. (2019). Tankyrase Disrupts Metabolic Homeostasis and Promotes Tumorigenesis by Inhibiting LKB1-AMPK Signalling. Nat. Commun. 10, 4363. 10.1038/s41467-019-12377-1 31554794 PMC6761205

[B10] Li N. Zhang Y. Han X. Liang K. Wang J. Feng L. (2015). Poly-ADP Ribosylation of PTEN by Tankyrases Promotes PTEN Degradation and Tumor Growth. Genes Dev. 29, 157–170. 10.1101/gad.251785.114 25547115 PMC4298135

[B11] Liu Y. Lu T. Zhang C. Xu J. Xue Z. Busuttil R. W. (2019). Activation of YAP Attenuates Hepatic Damage and Fibrosis in Liver Ischemia-Reperfusion Injury. J. Hepatology 71, 719–730. 10.1016/j.jhep.2019.05.029 PMC6773499 31201834

[B12] Liu Z. Wang Y. Dou C. Xu M. Sun L. Wang L. (2018). Hypoxia-induced Up-Regulation of VASP Promotes Invasiveness and Metastasis of Hepatocellular Carcinoma. Theranostics 8, 4649–4663. 10.7150/thno.26789 30279729 PMC6160773

[B13] Llovet J. M. De Baere T. Kulik L. Haber P. K. Greten T. F. Meyer T. (2021a). Locoregional Therapies in the Era of Molecular and Immune Treatments for Hepatocellular Carcinoma. Nat. Rev. Gastroenterol. Hepatol. 18, 293–313. 10.1038/s41575-020-00395-0 33510460

[B14] Llovet J. M. Kelley R. K. Villanueva A. Singal A. G. Pikarsky E. Roayaie S. (2021b). Hepatocellular Carcinoma. Nat. Rev. Dis. Prim. 7, 6. 10.1038/s41572-020-00240-3 33479224 PMC13537433

[B15] Matsumoto Y. La Rose J. Lim M. Adissu H. A. Law N. Mao X. (2017). Ubiquitin Ligase RNF146 Coordinates Bone Dynamics and Energy Metabolism. J. Clin. Invest. 127, 2612–2625. 10.1172/jci92233 28581440 PMC5490759

[B16] Shen J. Yu Z. Li N. (2018). The E3 Ubiquitin Ligase RNF146 Promotes Colorectal Cancer by Activating the Wnt/β-Catenin Pathway via Ubiquitination of Axin1. Biochem. Biophysical Res. Commun. 503, 991–997. 10.1016/j.bbrc.2018.06.107 29932918

[B17] Shi Z. Liu R. Lu Q. Zeng Z. Liu Y. Zhao J. (2021). UBE2O Promotes Hepatocellular Carcinoma Cell Proliferation and Invasion by Regulating the AMPKα2/mTOR Pathway. Int. J. Med. Sci. 18, 3749–3758. 10.7150/ijms.63220 34790050 PMC8579295

[B18] Singleton D. C. Macann A. Wilson W. R. (2021). Therapeutic Targeting of the Hypoxic Tumour Microenvironment. Nat. Rev. Clin. Oncol. 18 (12), 751–772. 10.1038/s41571-021-00539-4 34326502

[B19] Sung H. Ferlay J. Siegel R. L. Laversanne M. Soerjomataram I. Jemal A. (2021). Global Cancer Statistics 2020: GLOBOCAN Estimates of Incidence and Mortality Worldwide for 36 Cancers in 185 Countries. CA A Cancer J. Clin. 71, 209–249. 10.3322/caac.21660 33538338

[B20] Tang Z. Kang B. Li C. Chen T. Zhang Z. (2019). GEPIA2: an Enhanced Web Server for Large-Scale Expression Profiling and Interactive Analysis. Nucleic Acids Res. 47, W556–W560. 10.1093/nar/gkz430 31114875 PMC6602440

[B21] Wang L. Sun L. Liu R. Mo H. Niu Y. Chen T. (2021). Long Non-coding RNA MAPKAPK5-AS1/PLAGL2/HIF-1α Signaling Loop Promotes Hepatocellular Carcinoma Progression. J. Exp. Clin. Cancer Res. 40, 72. 10.1186/s13046-021-01868-z 33596983 PMC7891009

[B22] Wang Y. Lyu Y. Tu K. Xu Q. Yang Y. Salman S. (2021). Histone Citrullination by PADI4 Is Required for HIF-dependent Transcriptional Responses to Hypoxia and Tumor Vascularization. Sci. Adv. 7, eabe3771. 10.1126/sciadv.abe3771 34452909 PMC8397272

[B23] Zeng Z. Shi Z. Liu Y. Zhao J. Lu Q. Guo J. (2021). HIF-1α-activated TM4SF1-AS1 Promotes the Proliferation, Migration, and Invasion of Hepatocellular Carcinoma Cells by Enhancing TM4SF1 Expression. Biochem. Biophysical Res. Commun. 566, 80–86. 10.1016/j.bbrc.2021.06.011 34118595

[B24] Zhang Y. Liu S. Mickanin C. Feng Y. Charlat O. Michaud G. A. (2011). RNF146 Is a poly(ADP-Ribose)-Directed E3 Ligase that Regulates Axin Degradation and Wnt Signalling. Nat. Cell Biol. 13, 623–629. 10.1038/ncb2222 21478859

[B25] Zhou B. Yan J. Guo L. Zhang B. Liu S. Yu M. (2020). Hepatoma Cell-Intrinsic TLR9 Activation Induces Immune Escape through PD-L1 Upregulation in Hepatocellular Carcinoma. Theranostics 10, 6530–6543. 10.7150/thno.44417 32483468 PMC7255037

