# Supplementary material for: HIF-1/2α-Activated RNF146 Enhances the Proliferation and Glycolysis of Hepatocellular Carcinoma Cells via the PTEN/AKT/mTOR Pathway
Source: Front Cell Dev Biol. 2022 May 27;10:893888. doi: 10.3389/fcell.2022.893888 (PMC9200061; doi:10.3389/fcell.2022.893888)
Supplement: Supplementary file 1 [file Table1.DOCX]

**Supplementary Table 1 The top ten pathways of RNF146 revealed by GSEA analysis of the TCGA-LIHC dataset**

| GENESET | ES | NES | NP | FDR | FWER |
| --- | --- | --- | --- | --- | --- |
| KEGG_UBIQUITIN_MEDIATED_PROTEOLYSIS | 0.6927 | 2.1959 | 0 | 0 | 0 |
| KEGG_CHRONIC_MYELOID_LEUKEMIA | 0.6973 | 2.1369 | 0 | 0.0029 | 0.001 |
| KEGG_ERBB_SIGNALING_PATHWAY | 0.6463 | 2.0886 | 0 | 0.0067 | 0.004 |
| KEGG_GNRH_SIGNALING_PATHWAY | 0.6183 | 2.0716 | 0 | 0.005 | 0.004 |
| KEGG_INOSITOL_PHOSPHATE_METABOLISM | 0.6842 | 2.0649 | 0 | 0.0043 | 0.005 |
| KEGG_REGULATION_OF_AUTOPHAGY | 0.6623 | 2.0538 | 0 | 0.0047 | 0.007 |
| KEGG_MTOR_SIGNALING_PATHWAY | 0.6635 | 2.0493 | 0 | 0.0044 | 0.008 |
| KEGG_RNA_DEGRADATION | 0.6902 | 2.0476 | 0 | 0.0041 | 0.01 |
| KEGG_ENDOCYTOSIS | 0.6019 | 2.0412 | 0 | 0.0038 | 0.011 |
| KEGG_WNT_SIGNALING_PATHWAY | 0.6067 | 2.0402 | 0 | 0.0036 | 0.011 |
