# Supplementary material for: HIF-1/2α-Activated RNF146 Enhances the Proliferation and Glycolysis of Hepatocellular Carcinoma Cells via the PTEN/AKT/mTOR Pathway
Source: Front Cell Dev Biol. 2022 May 27;10:893888. doi: 10.3389/fcell.2022.893888 (PMC9200061; doi:10.3389/fcell.2022.893888)

Figure 1C

HIF-1α

HIF-2α

RNF146

β-tubulin

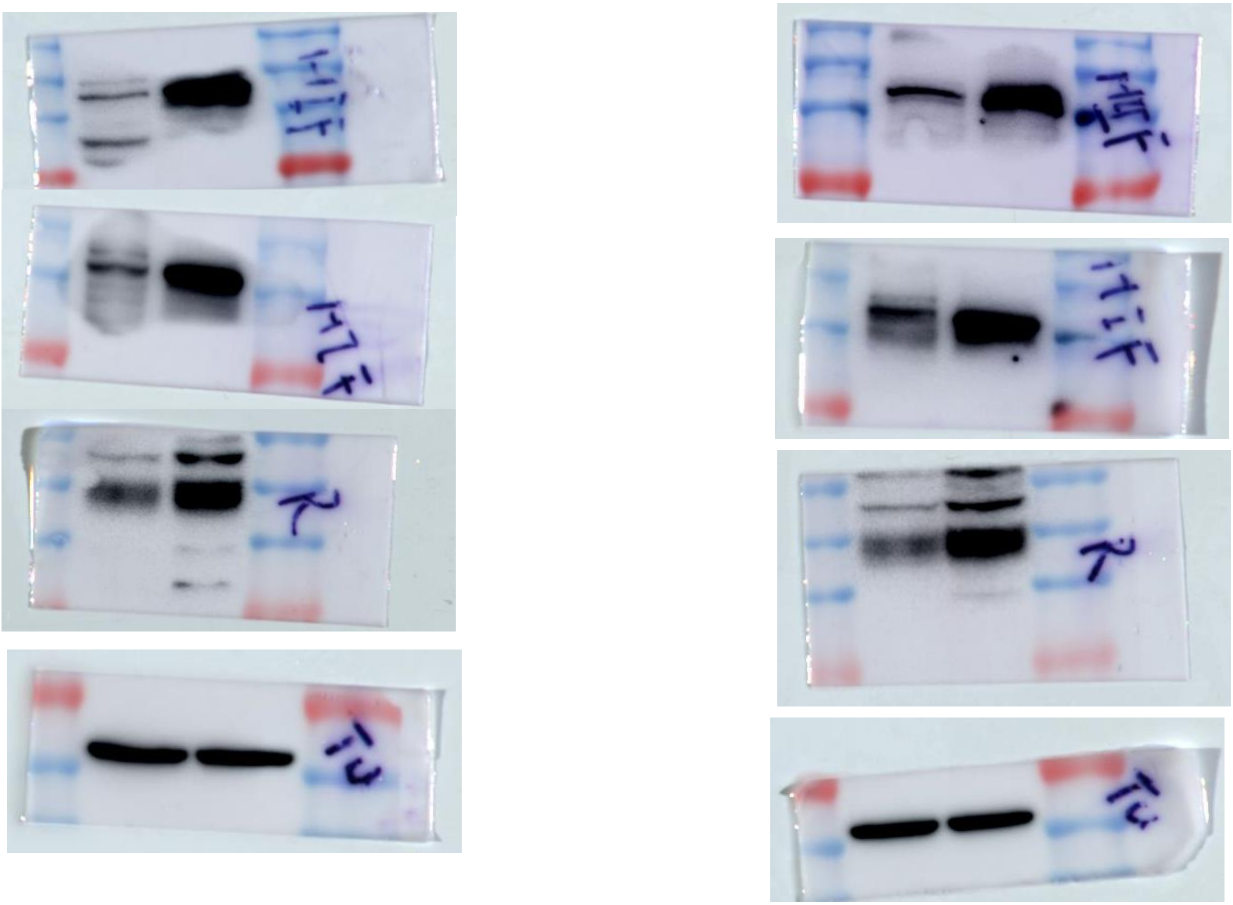

Hep3B

Huh7

Figure 1E

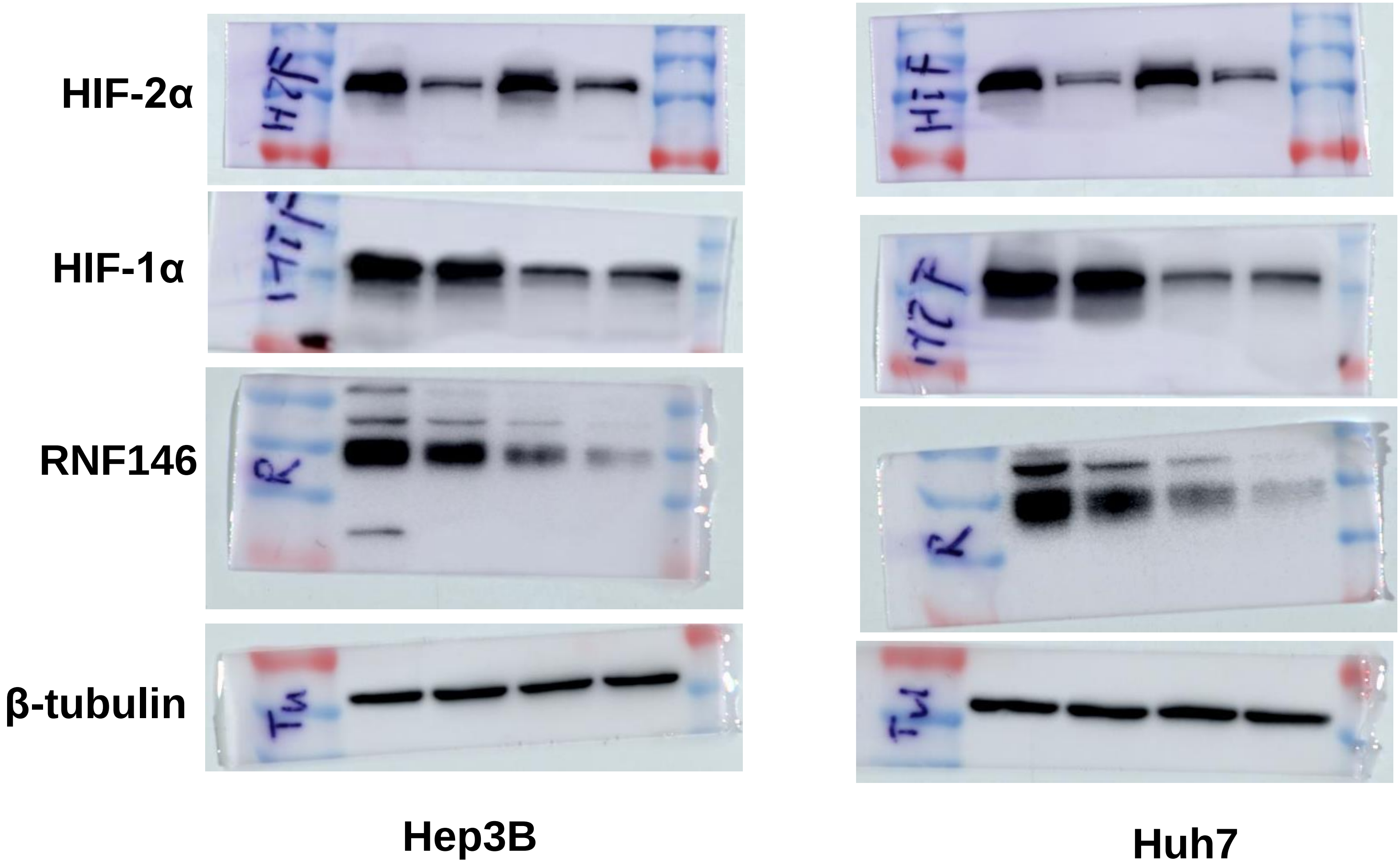

Figure 2D

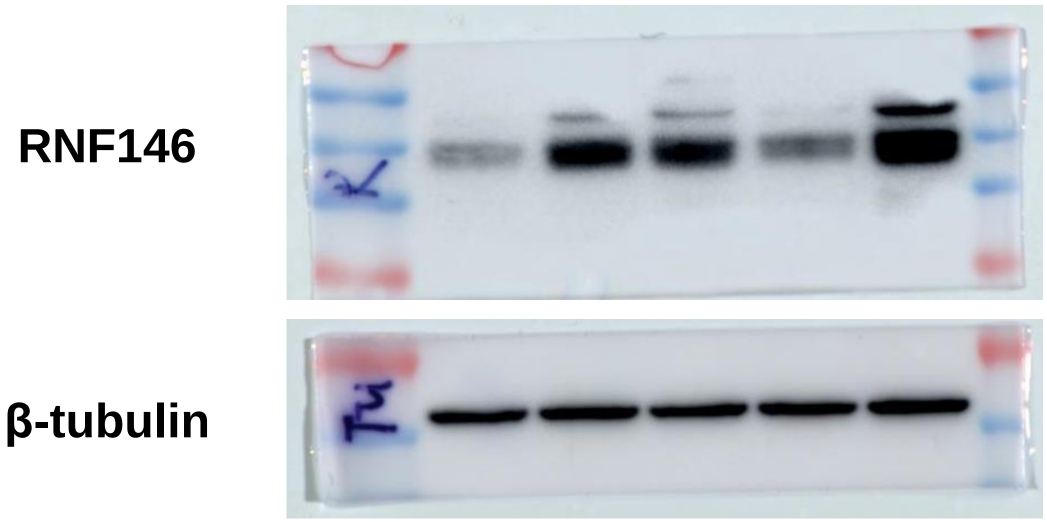

Figure 3A

RNF146

β-tubulin

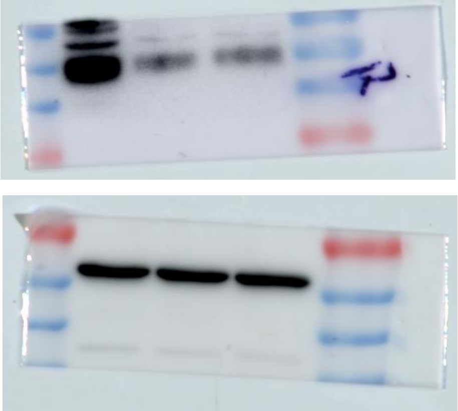

Figure 4A

RNF146

β-tubulin

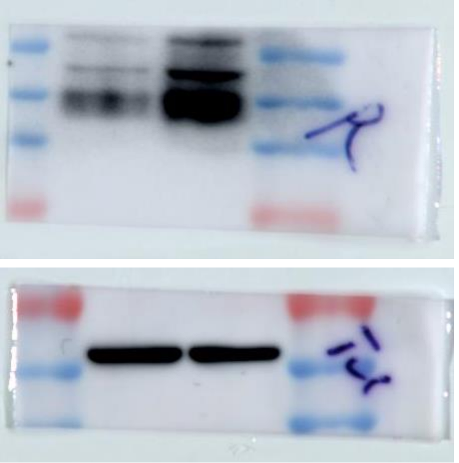

Figure 5D

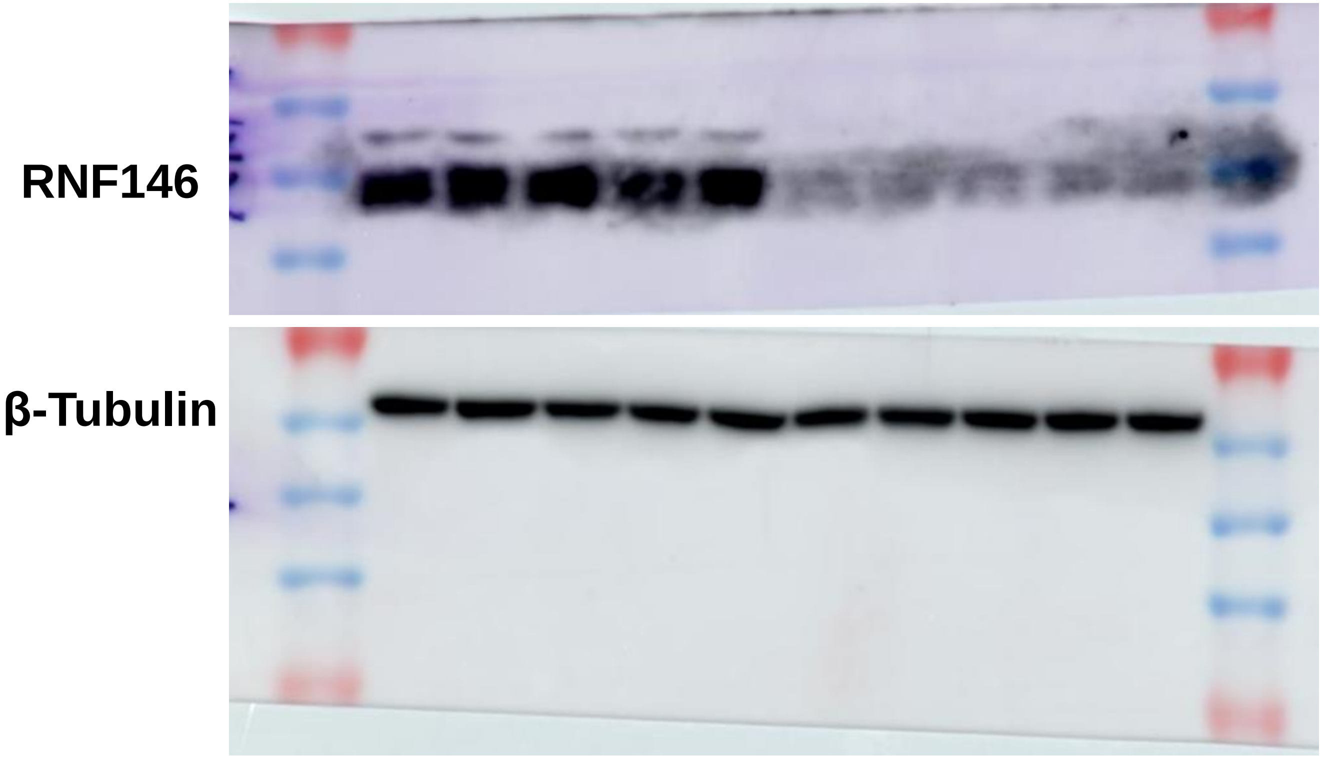

Figure 6B

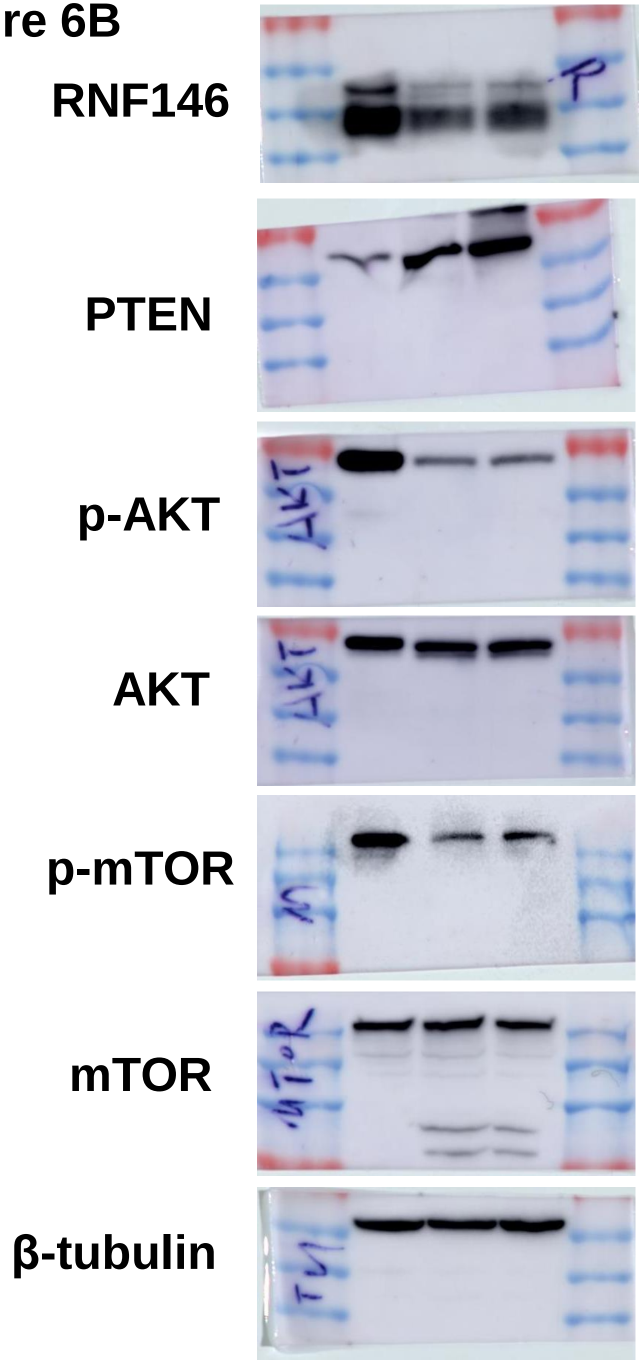

Figure 6C

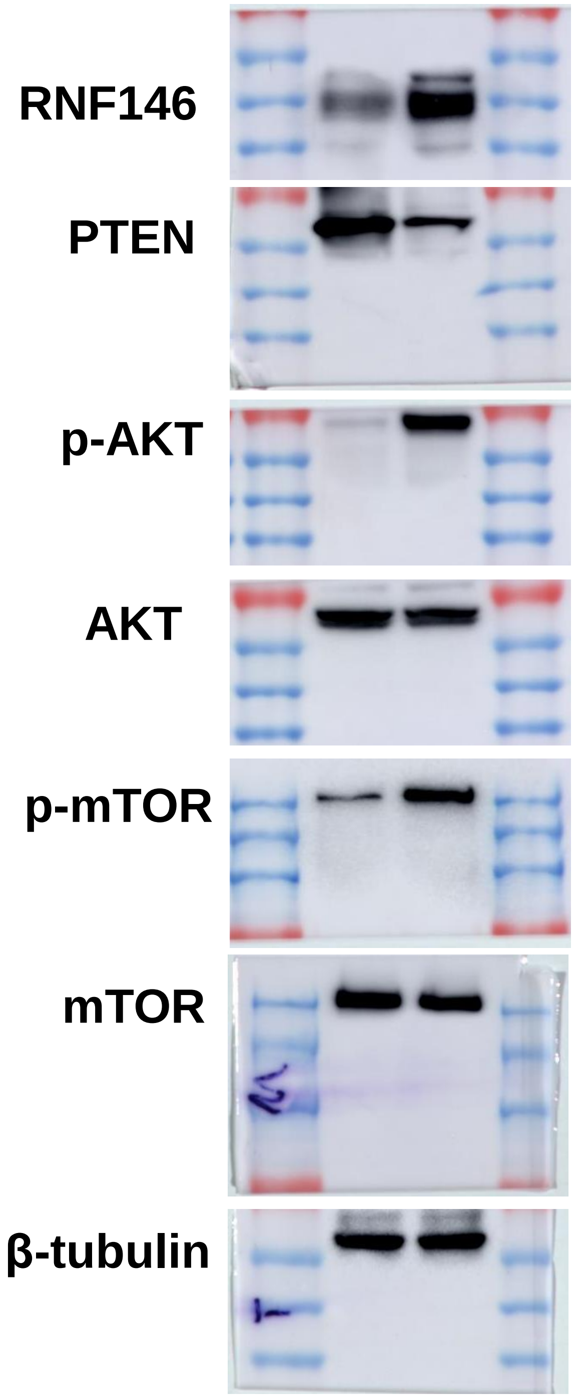

Figure 6D

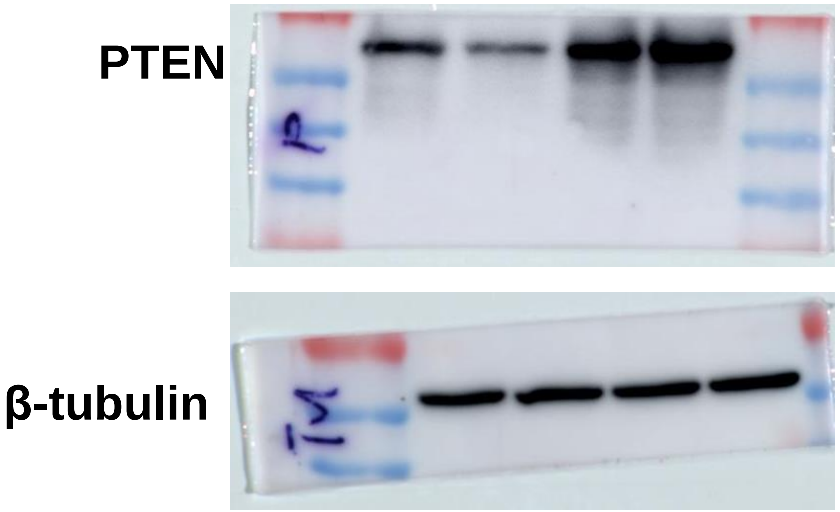

Figure 6E

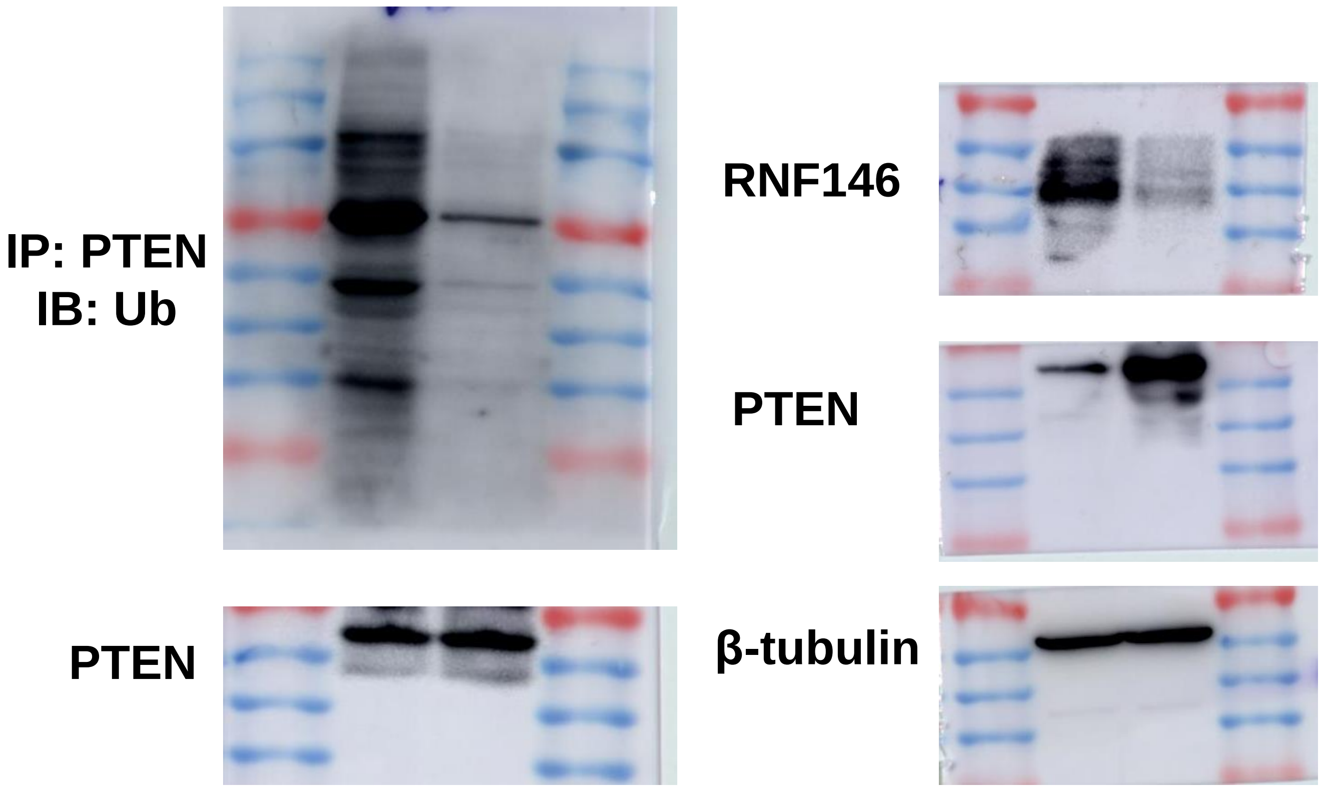

Figure 6F

PTEN

RNF146

β-tubulin

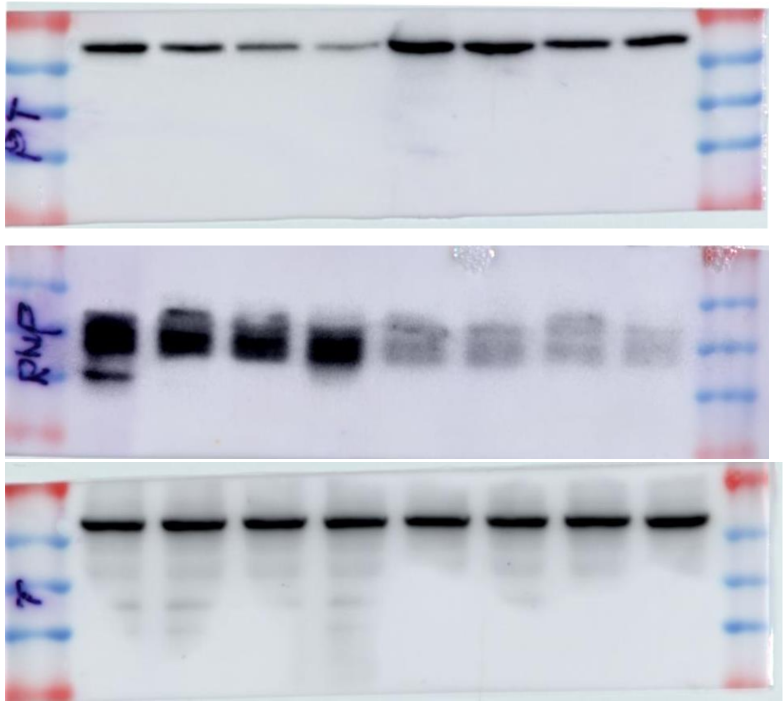

Figure 7A

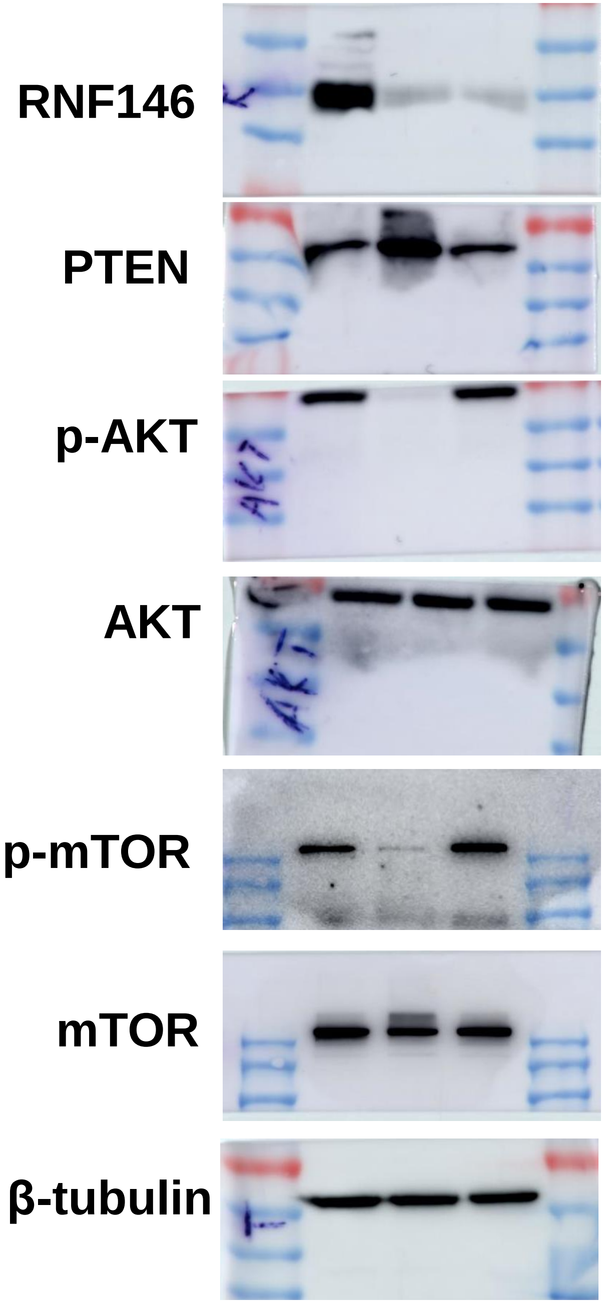

Supplement: Supplementary file 2 [file DataSheet1.ZIP › WB data.pdf]
