# Supplementary figures and images for: HIF-1/2α-Activated RNF146 Enhances the Proliferation and Glycolysis of Hepatocellular Carcinoma Cells via the PTEN/AKT/mTOR Pathway
Source: Front Cell Dev Biol. 2022 May 27;10:893888. doi: 10.3389/fcell.2022.893888 (PMC9200061; doi:10.3389/fcell.2022.893888)

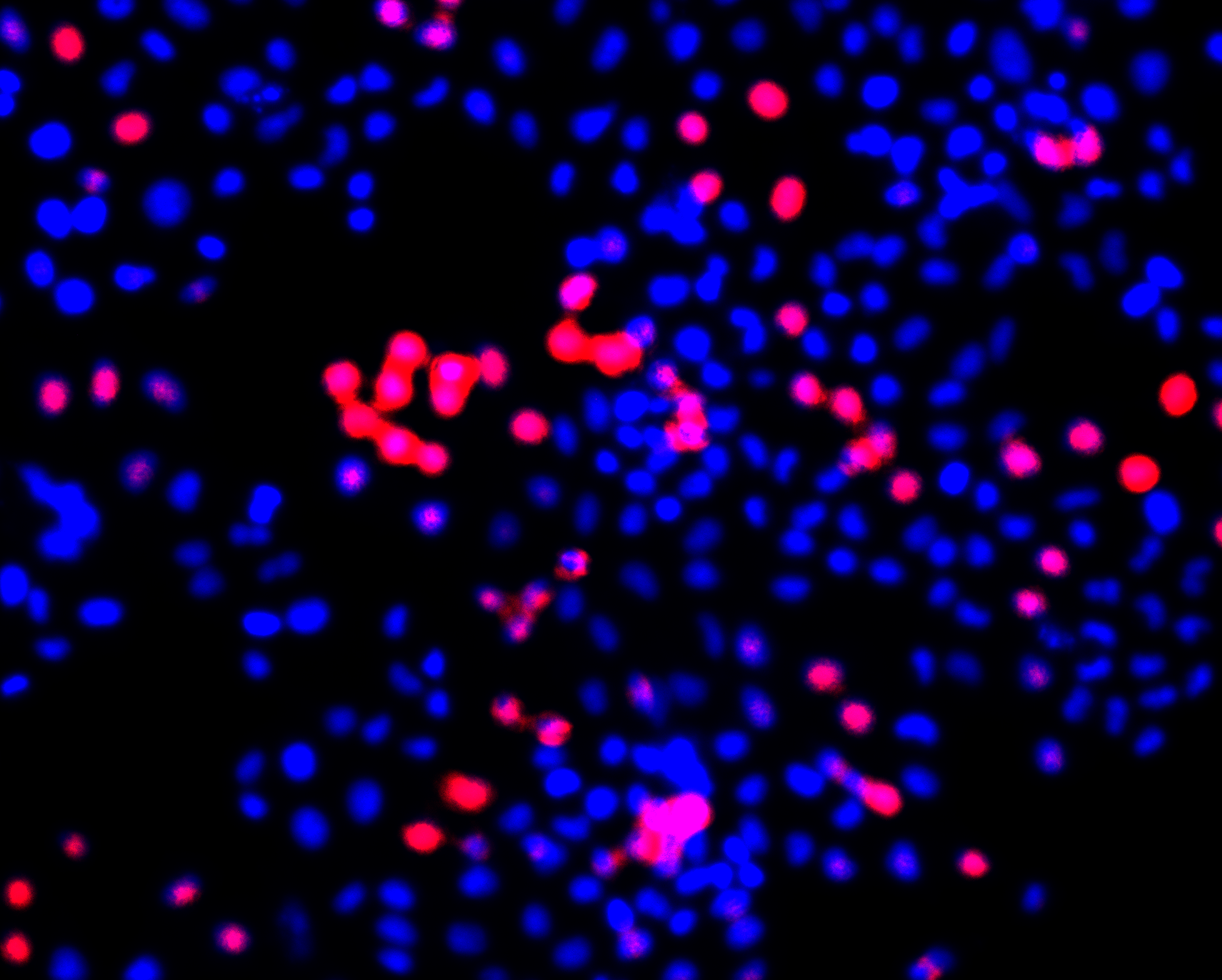

Supplement: Supplementary file 2 [file DataSheet1.ZIP › Figure 4C (2).tif]

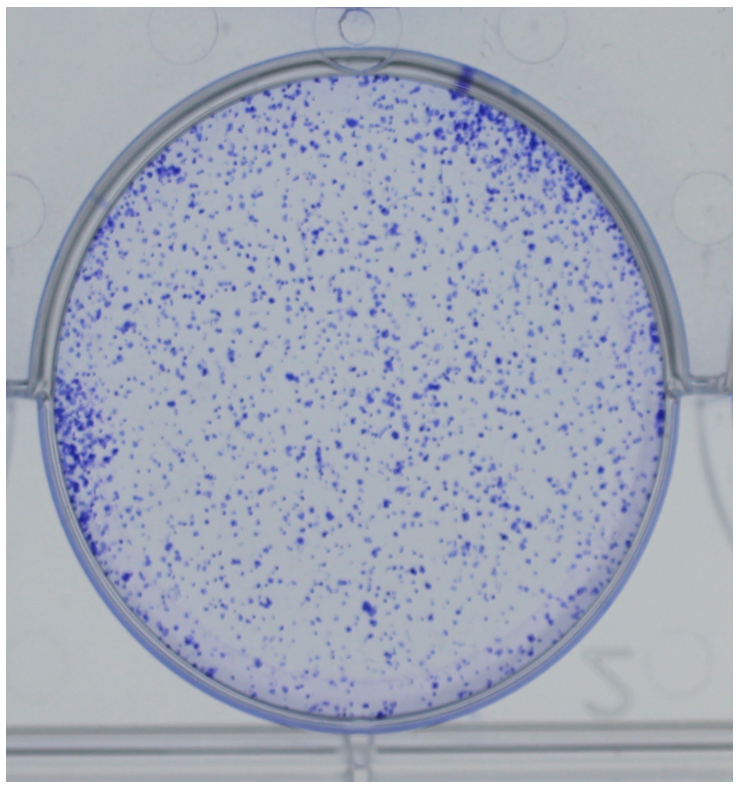

Supplement: Supplementary file 2 [file DataSheet1.ZIP › Figure 4D (1).png]

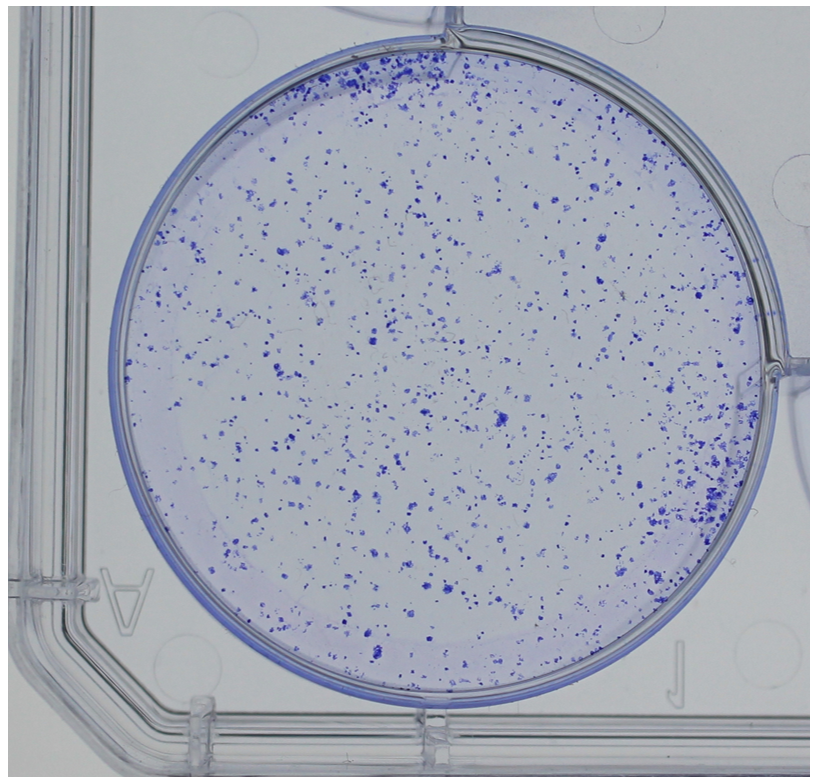

Supplement: Supplementary file 2 [file DataSheet1.ZIP › Figure 4D (2).png]

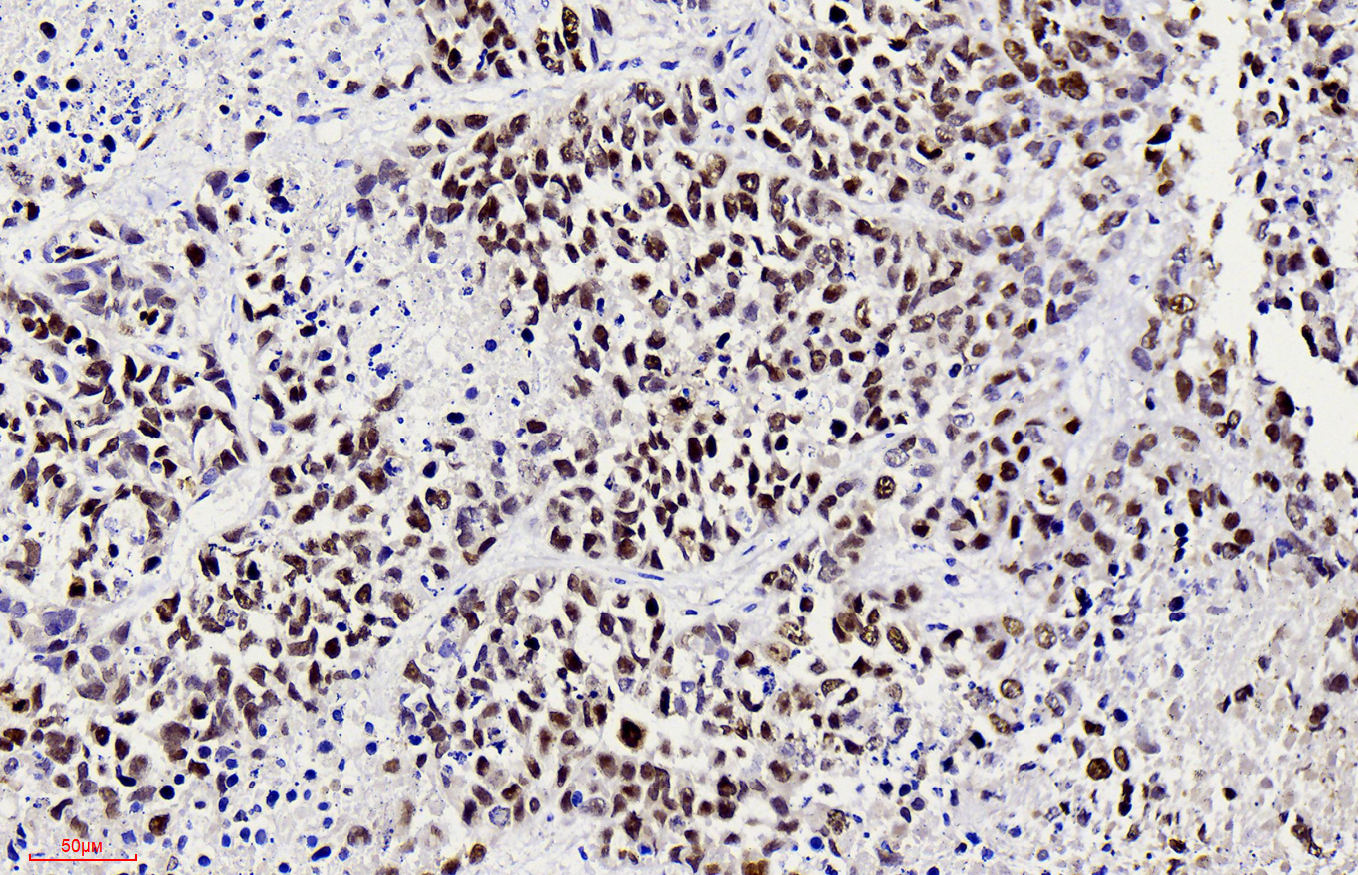

Supplement: Supplementary file 2 [file DataSheet1.ZIP › Figure 5C (1).png]

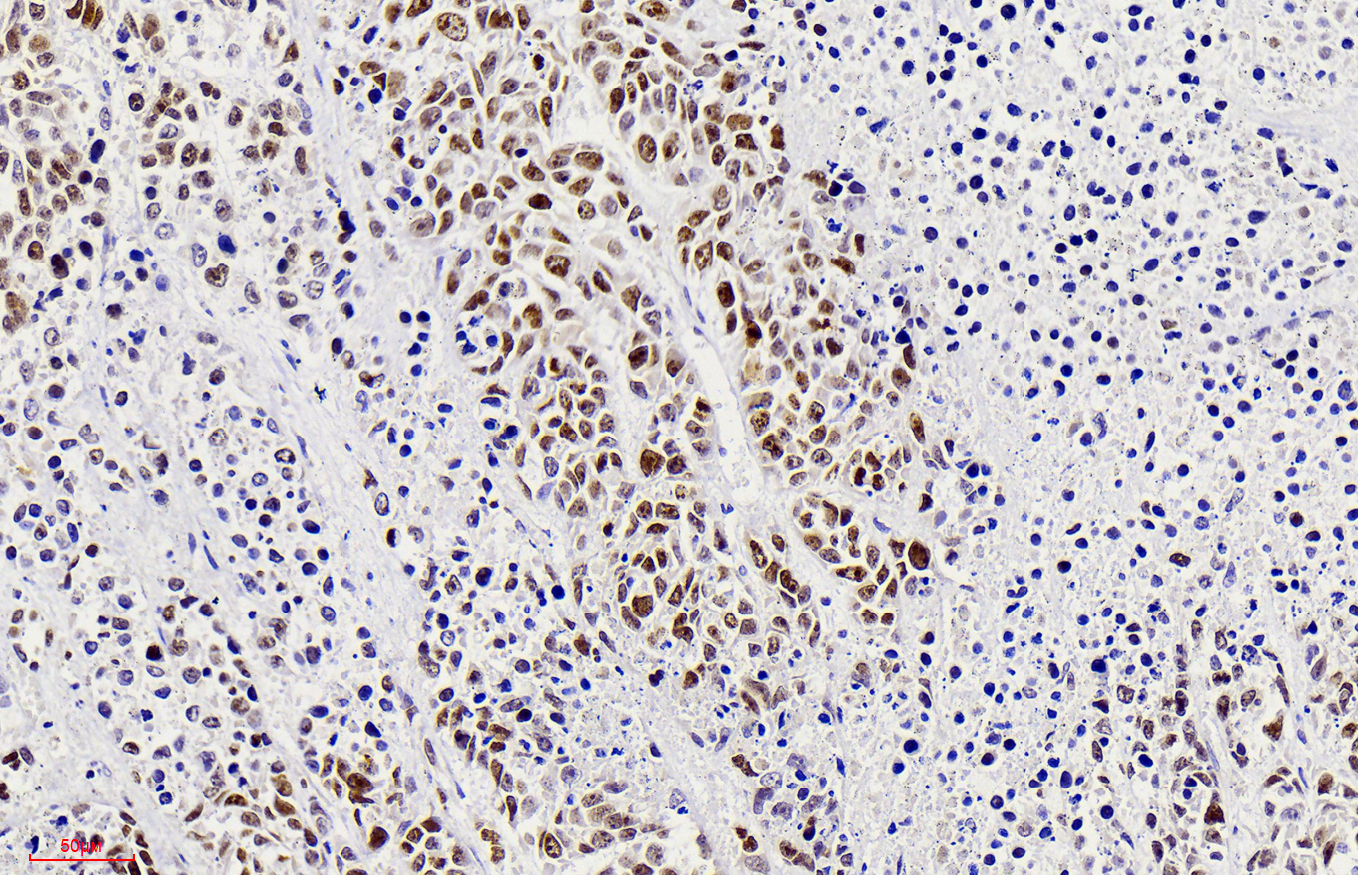

Supplement: Supplementary file 2 [file DataSheet1.ZIP › Figure 5C (2).png]

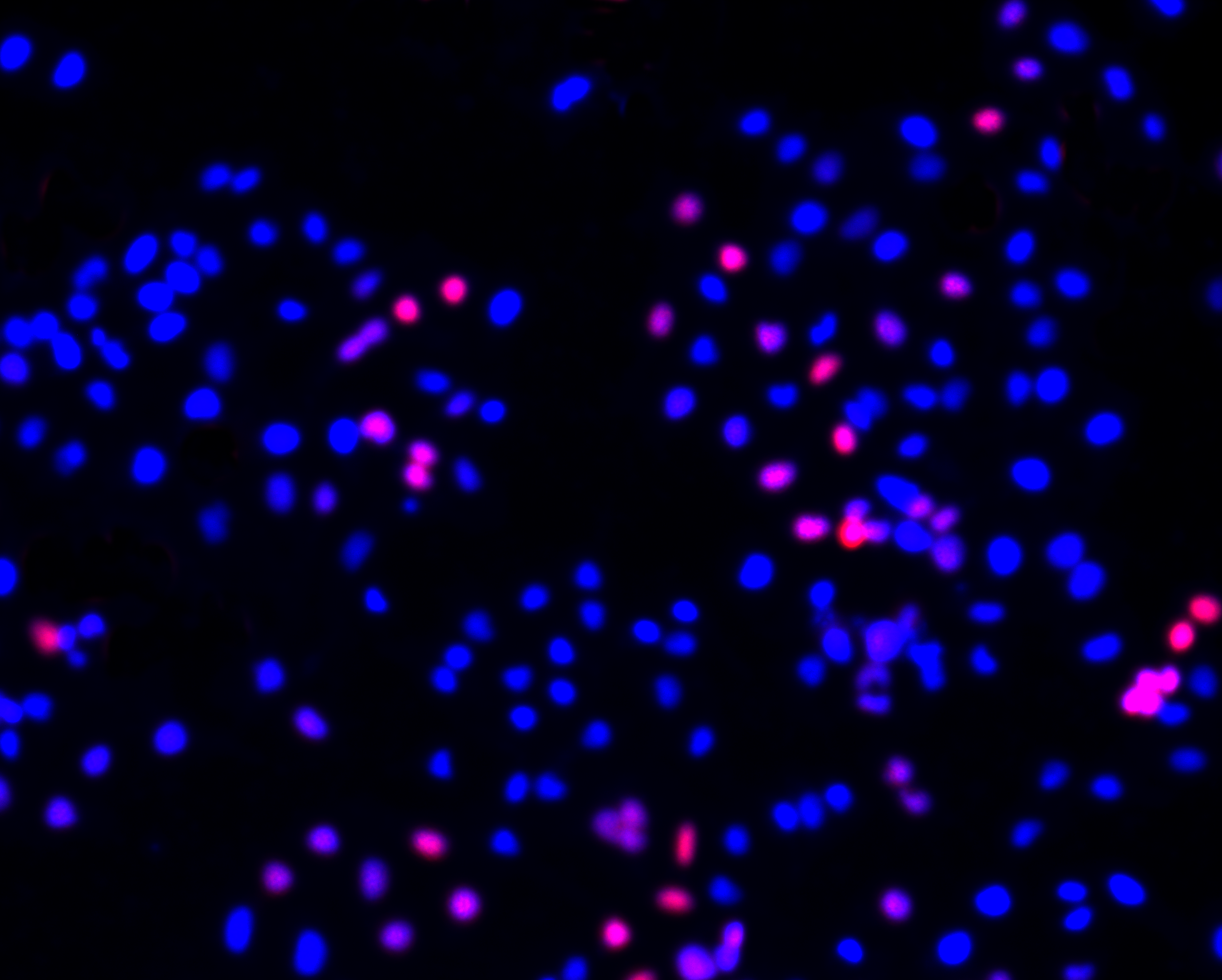

Supplement: Supplementary file 2 [file DataSheet1.ZIP › Figure 7C (1).tif]

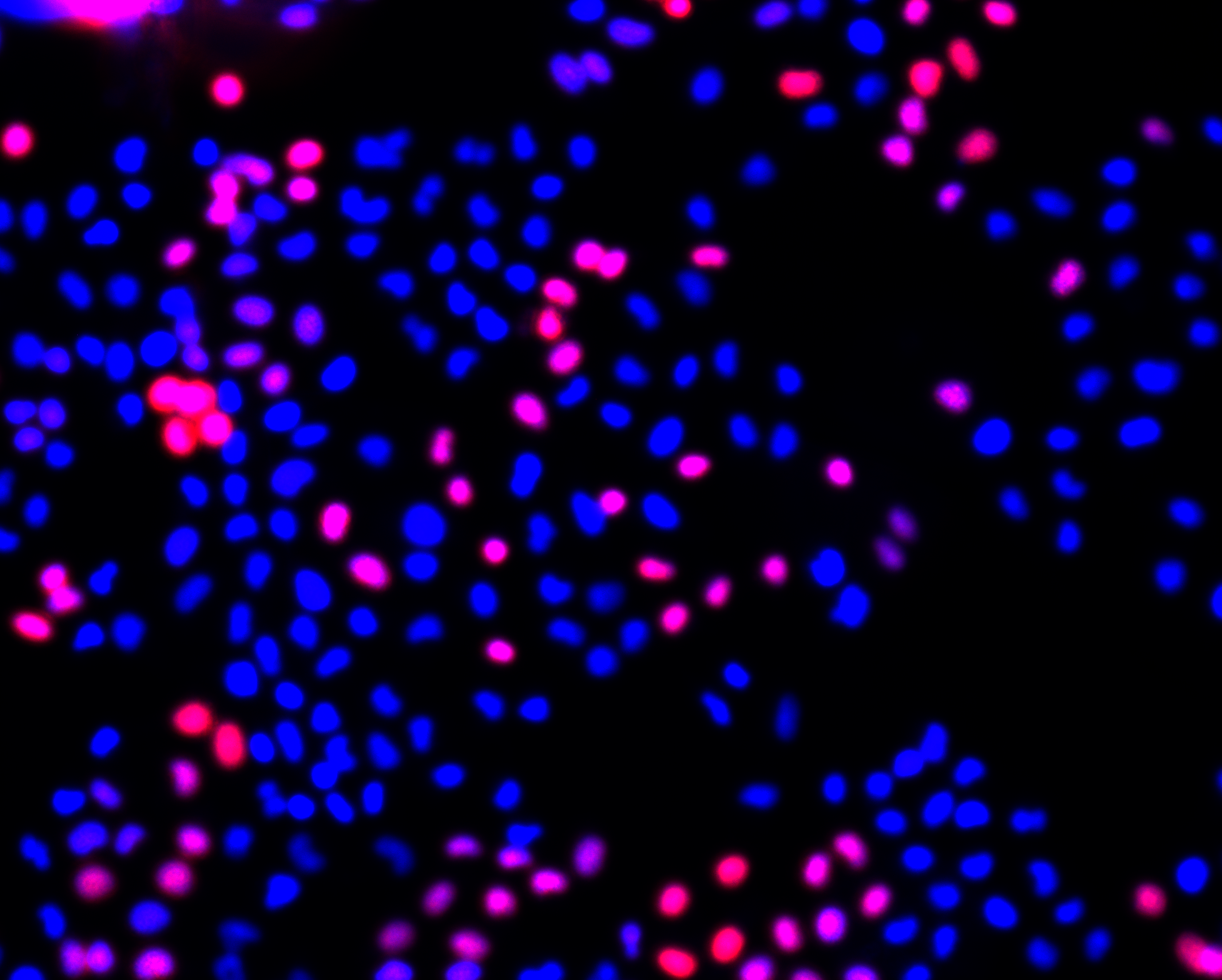

Supplement: Supplementary file 2 [file DataSheet1.ZIP › Figure 7C (2).tif]

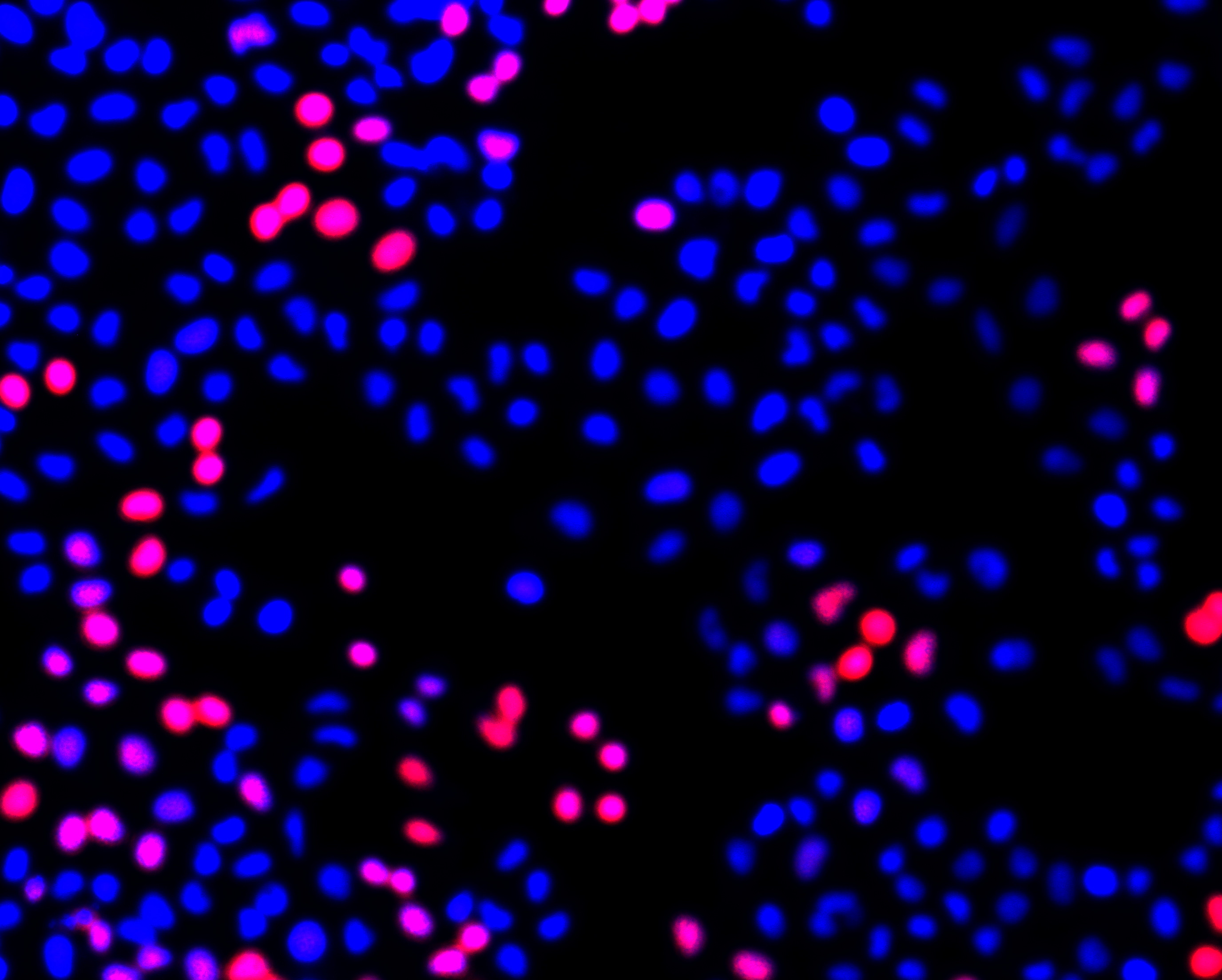

Supplement: Supplementary file 2 [file DataSheet1.ZIP › Figure 7C (3).tif]

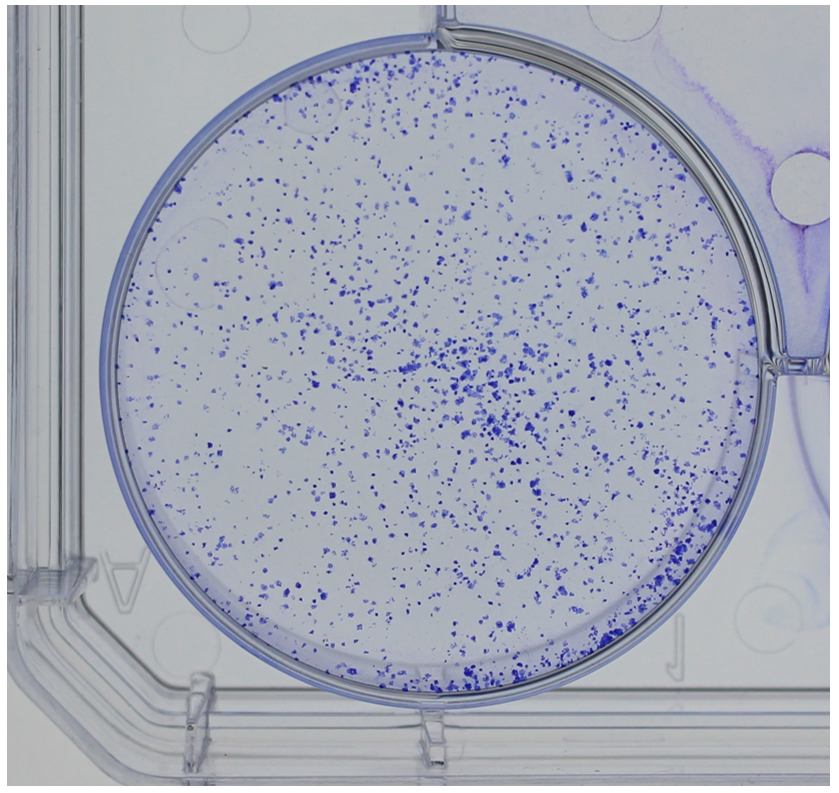

Supplement: Supplementary file 2 [file DataSheet1.ZIP › Figure 7D (1).png]

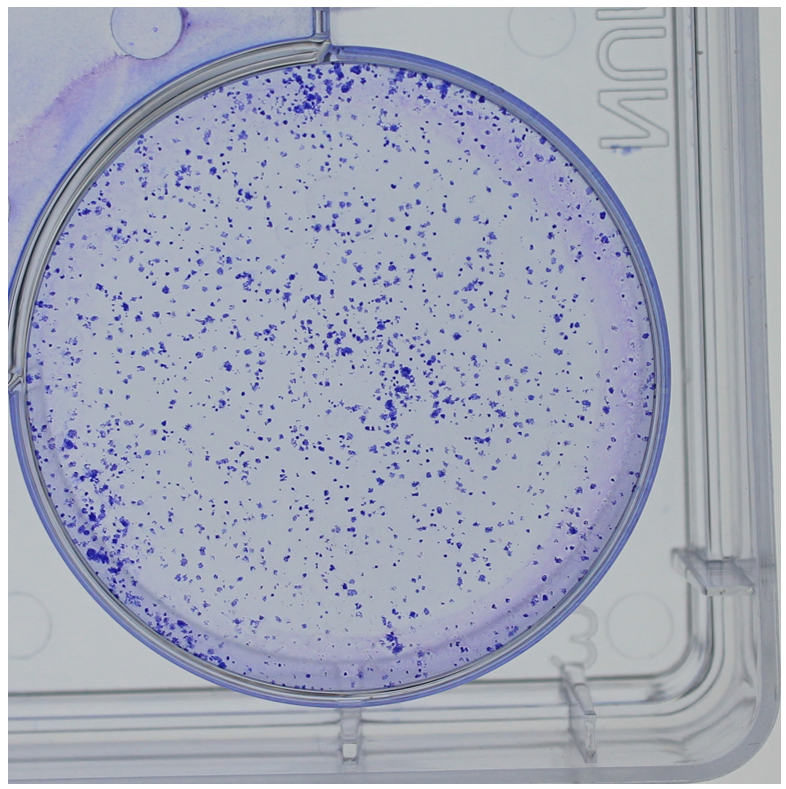

Supplement: Supplementary file 2 [file DataSheet1.ZIP › Figure 7D (2).png]

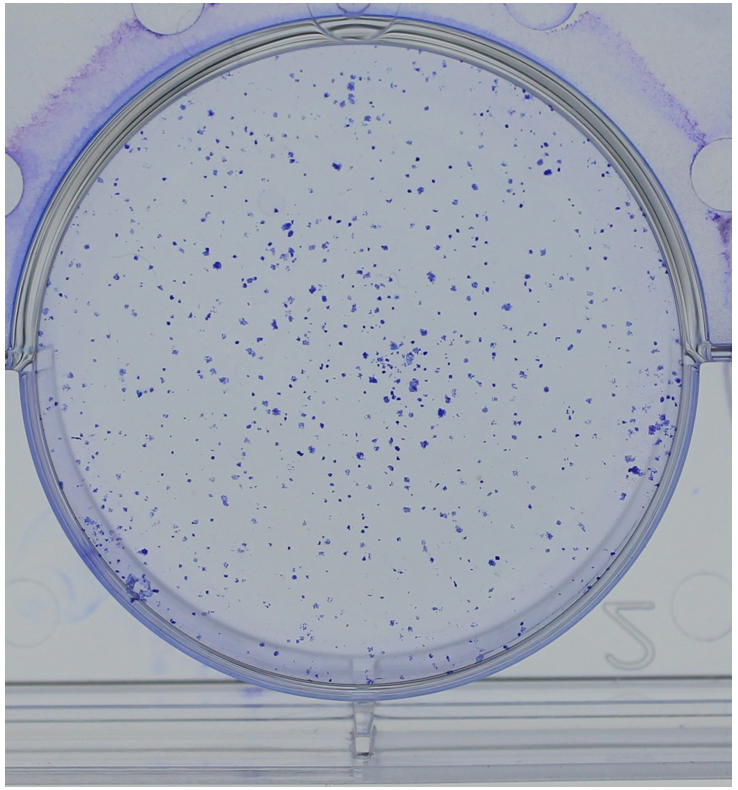

Supplement: Supplementary file 2 [file DataSheet1.ZIP › Figure 7D (3).png]

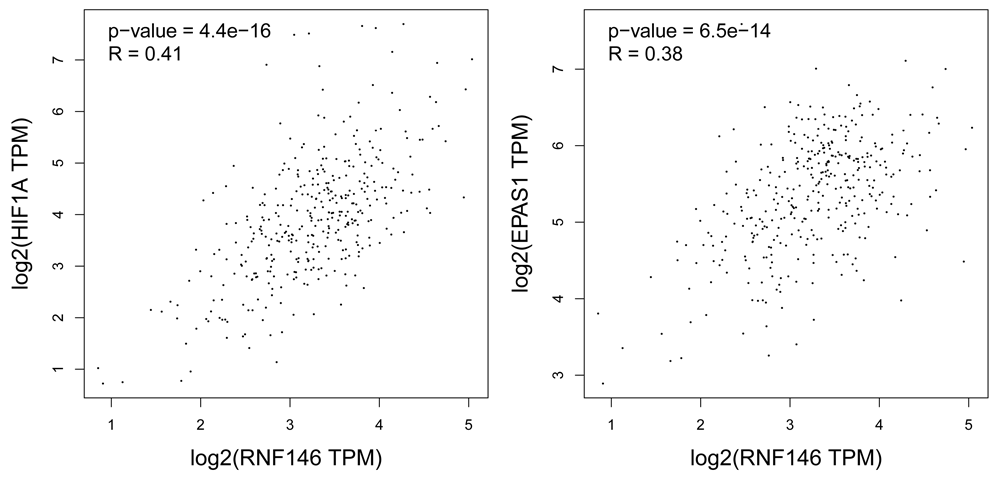

Supplement: Supplementary file 3 [file Image1.TIF]

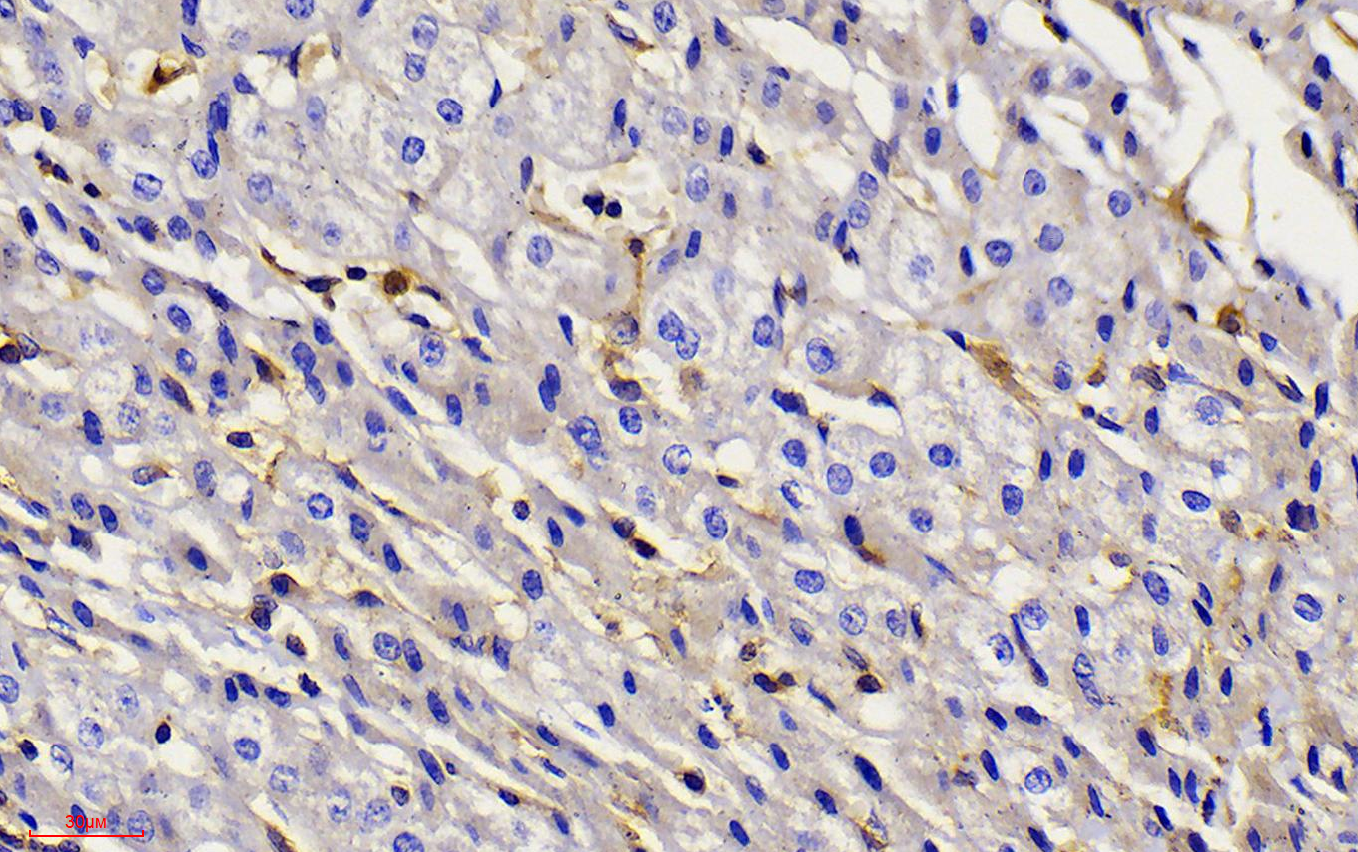

Supplement: Supplementary file 5 [file DataSheet2.ZIP › Figure 2B (1).png]

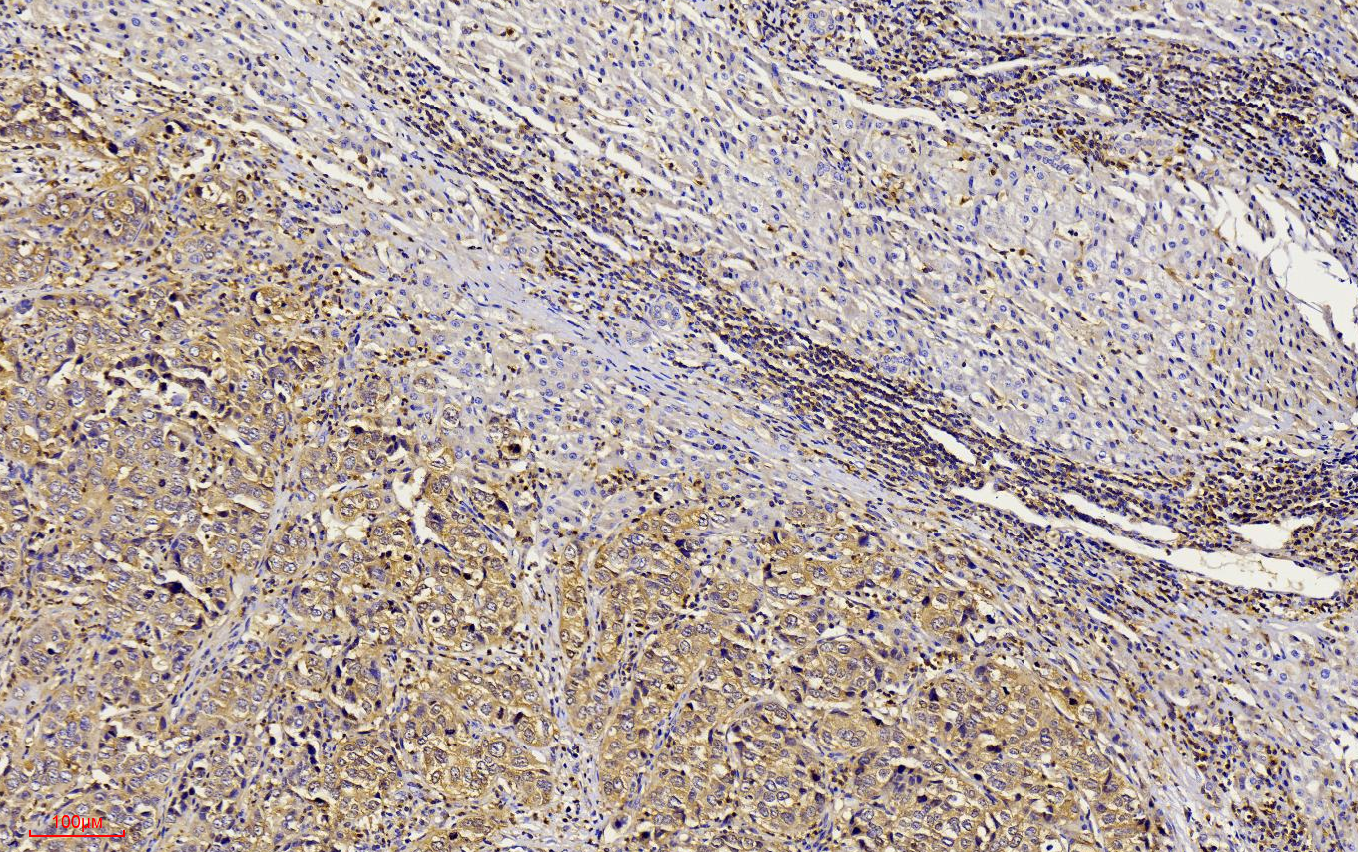

Supplement: Supplementary file 5 [file DataSheet2.ZIP › Figure 2B (2).png]

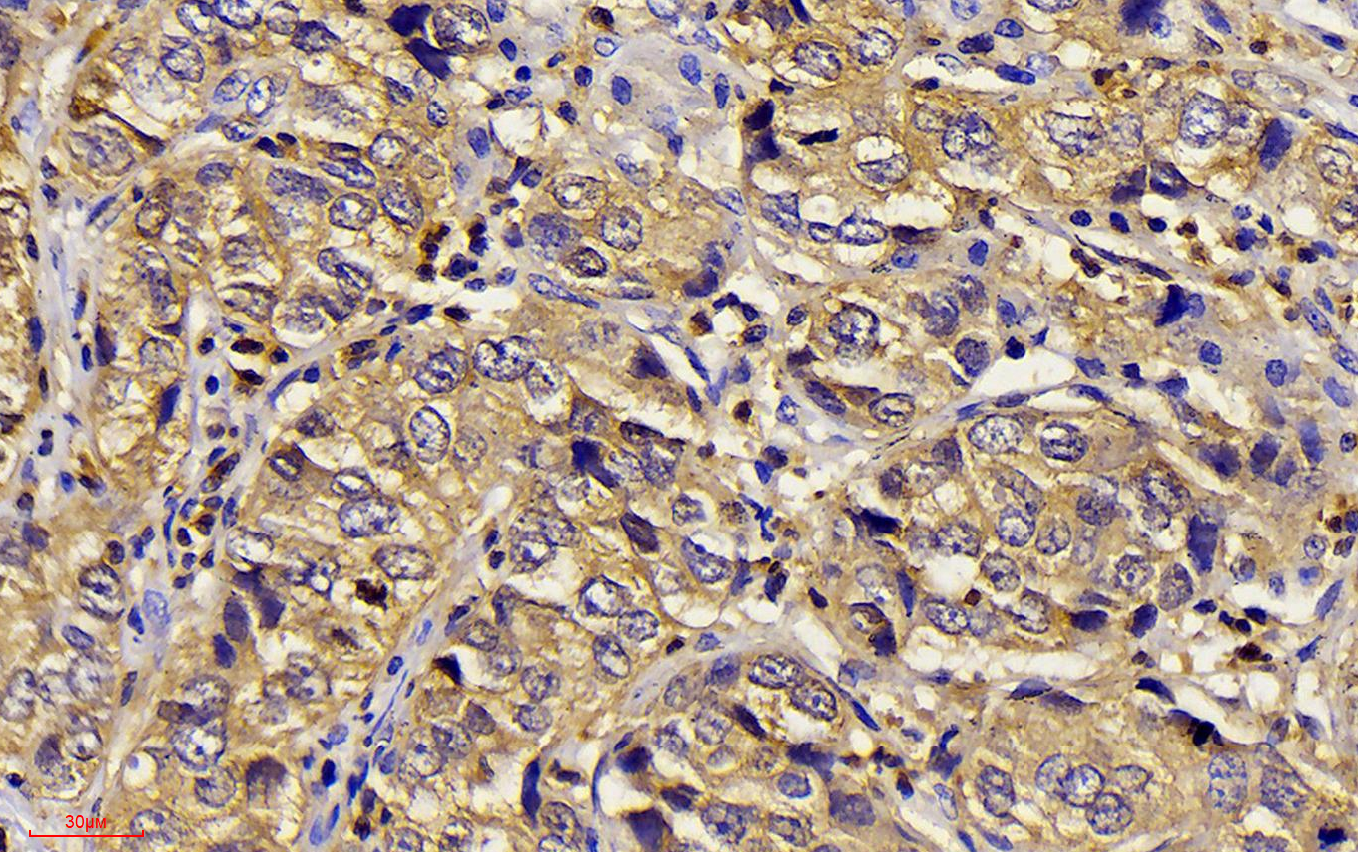

Supplement: Supplementary file 5 [file DataSheet2.ZIP › Figure 2B (3).png]

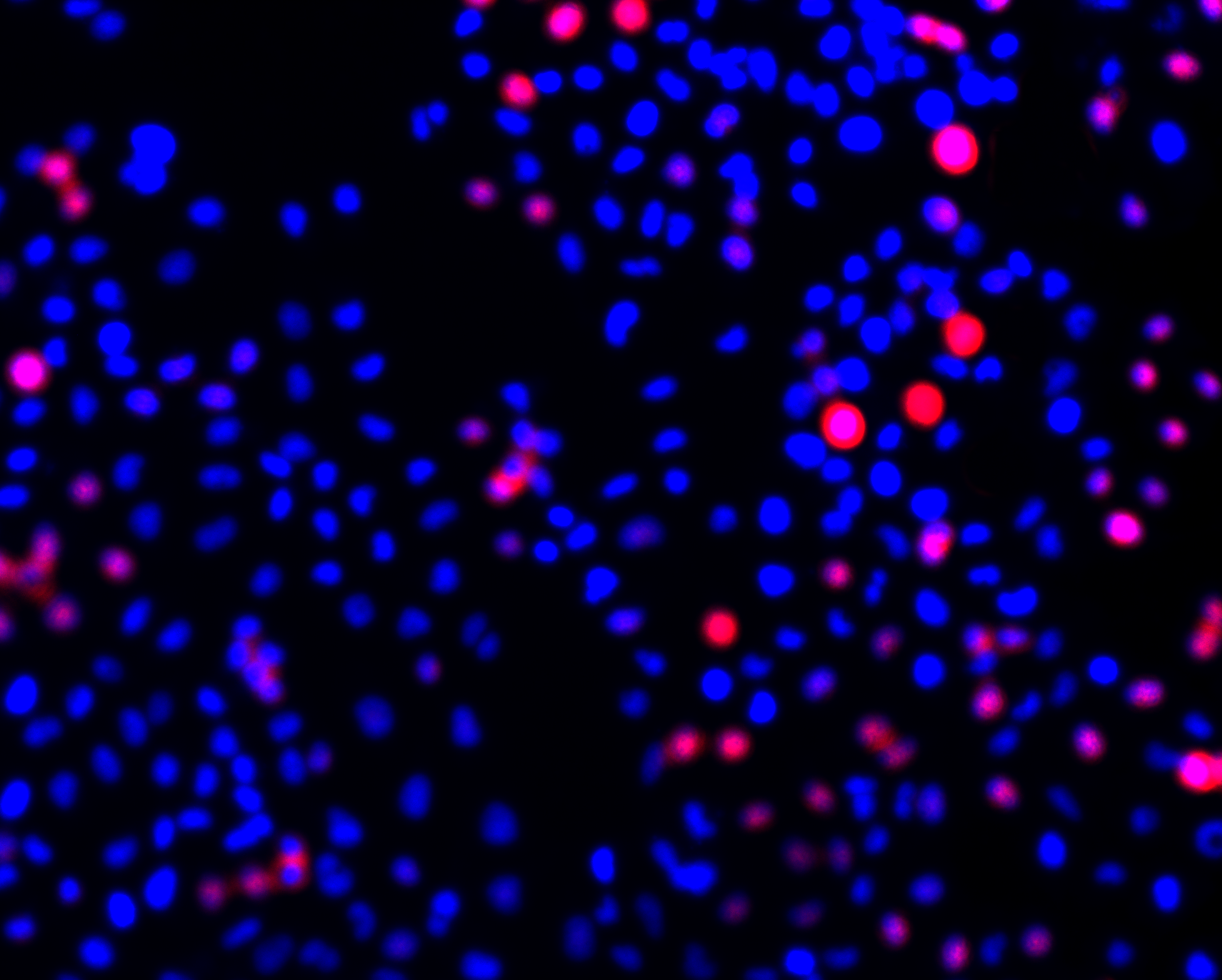

Supplement: Supplementary file 5 [file DataSheet2.ZIP › Figure 3C (1).tif]

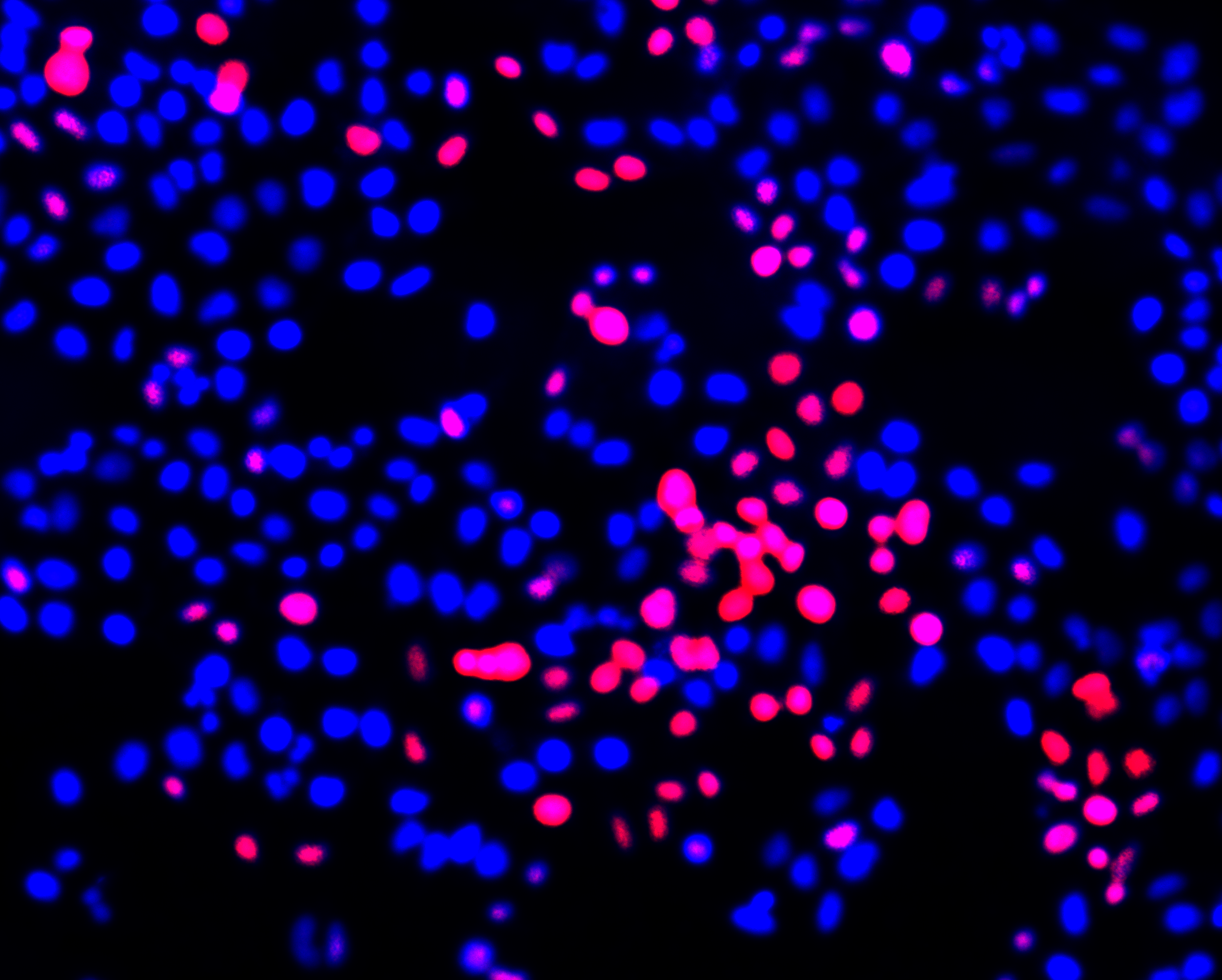

Supplement: Supplementary file 5 [file DataSheet2.ZIP › Figure 3C (2).tif]

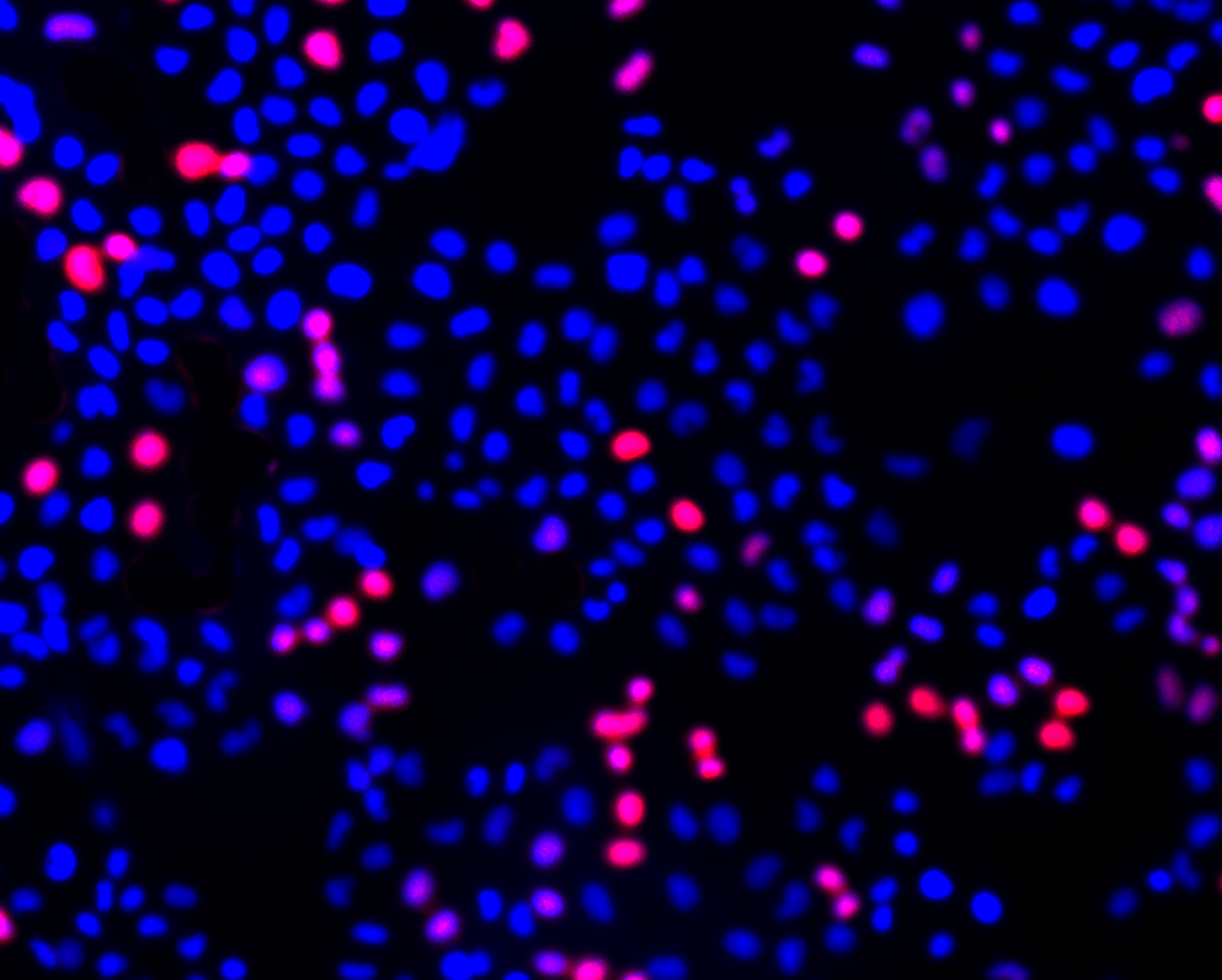

Supplement: Supplementary file 5 [file DataSheet2.ZIP › Figure 3C (3).tif]

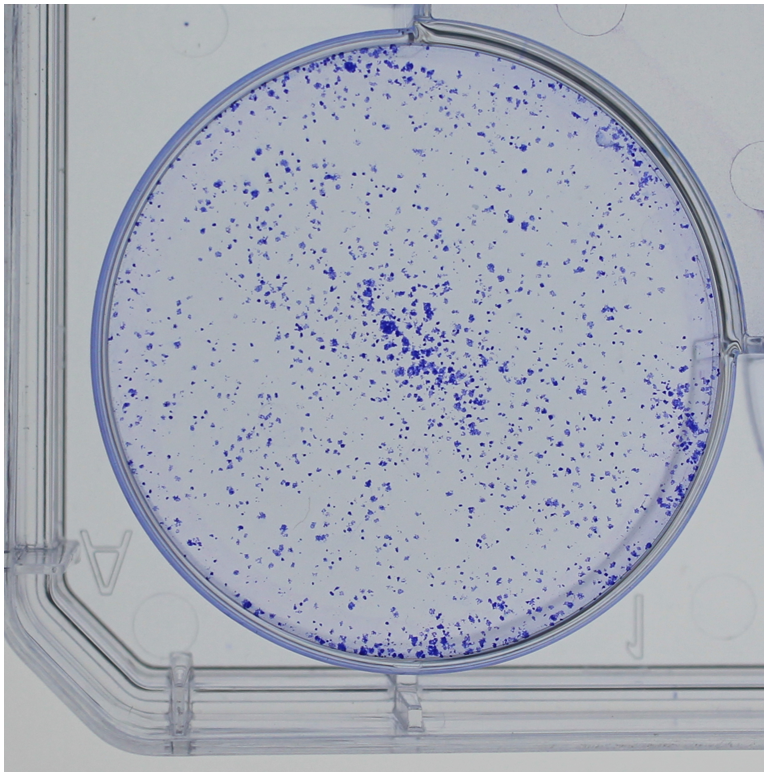

Supplement: Supplementary file 5 [file DataSheet2.ZIP › Figure 3D (1).png]

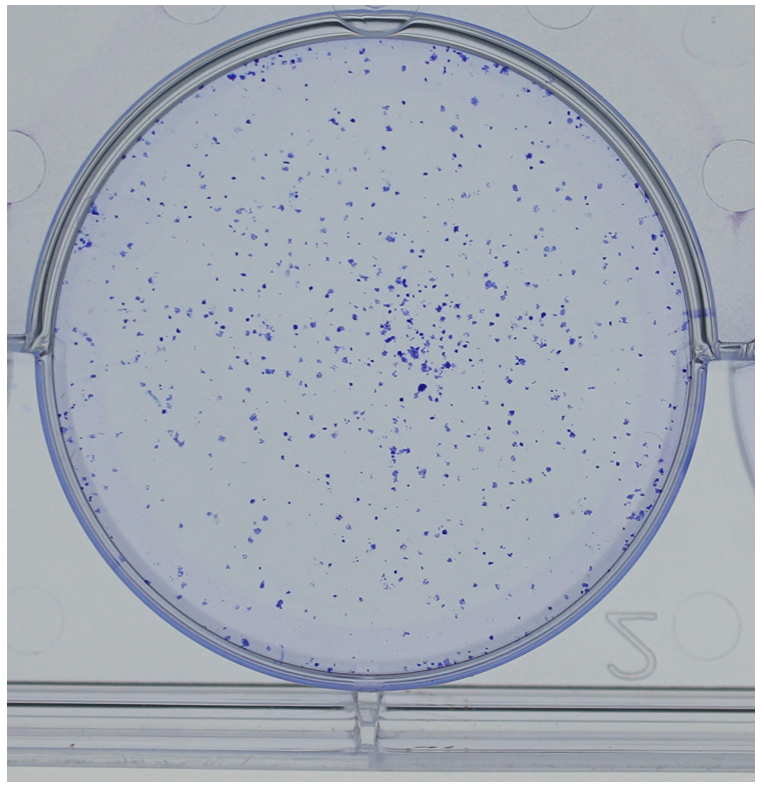

Supplement: Supplementary file 5 [file DataSheet2.ZIP › Figure 3D (2).png]

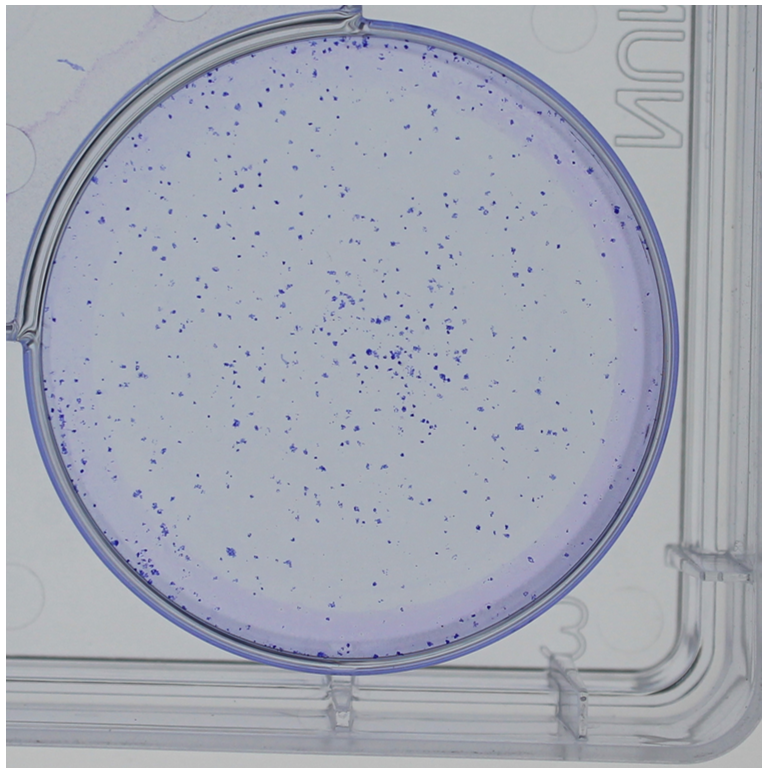

Supplement: Supplementary file 5 [file DataSheet2.ZIP › Figure 3D (3).png]

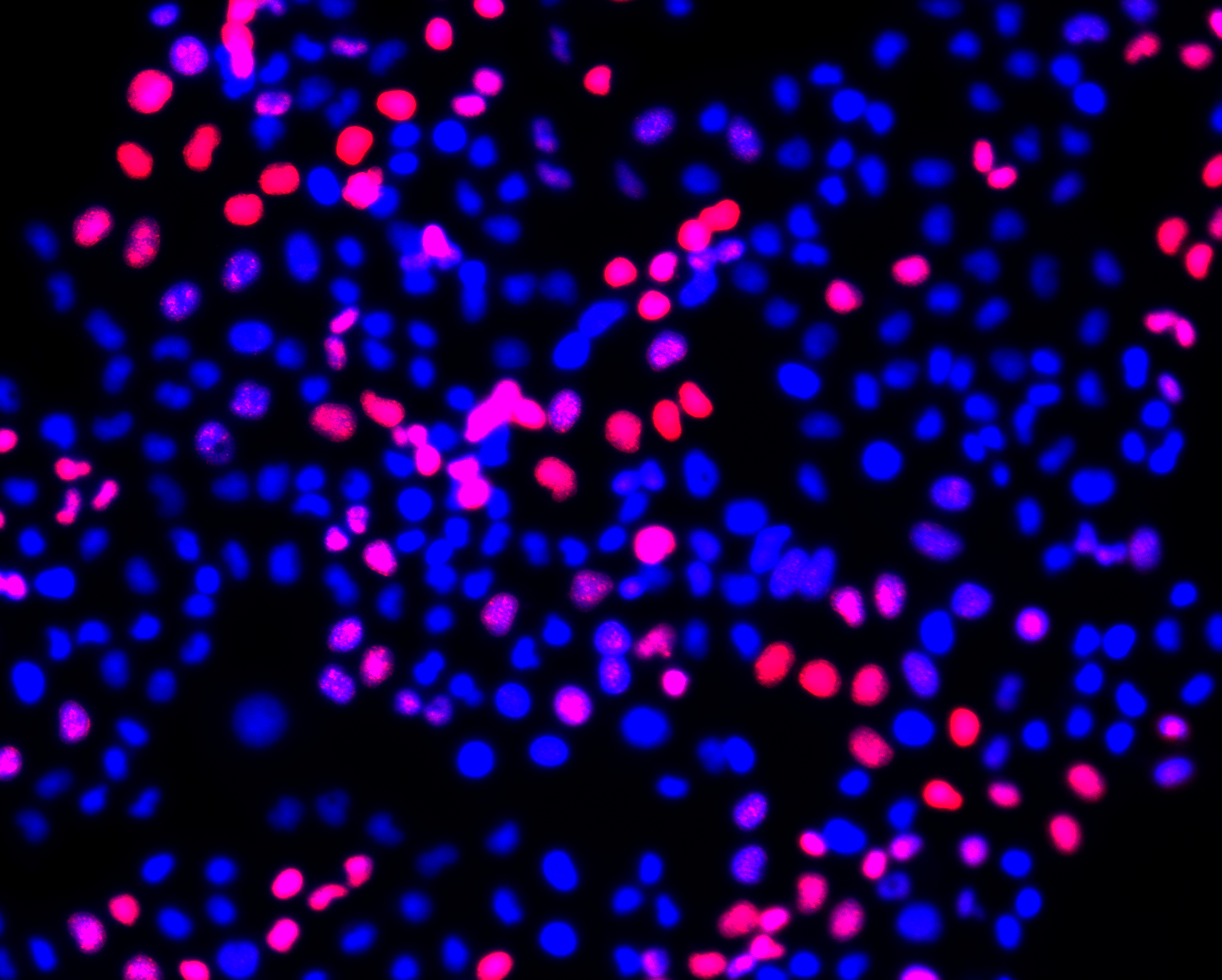

Supplement: Supplementary file 5 [file DataSheet2.ZIP › Figure 4C (1).tif]
